# Biallelic mutations in *IQCN*, encoding a novel acroplaxome protein, lead to fertilization failure and male infertility with defects in the acrosome and shaping of the spermatid head in humans and mice

**DOI:** 10.1093/lifemedi/lnac050

**Published:** 2022-11-04

**Authors:** Yanwei Sha, Yongjie Chen, Xiong Wang, Ranran Meng, Xiaoyan Yang, Youzhu Li, Pengpeng Jin, Shanze Li, Jie Chen, Tianyu Shao, Dan Xu, Yibiao Guo, Zhaodi Jiang, Yuhua Li, Shuntai Yu, Lin Li, Fengchao Wang

**Affiliations:** Department of Andrology, Women and Children’s Hospital, School of Medicine, Xiamen University, Xiamen 361005, China; Fujian Provincial Key Laboratory of Reproductive Health Research, School of Medicine, Xiamen University, Xiamen 361102, China; State Key Laboratory of Molecular Vaccinology and Molecular Diagnostics, School of Public Health, Xiamen University, Xiamen 361102, China; Central Laboratory, Beijing Obstetrics and Gynecology Hospital, Capital Medical University, Beijing Maternal and Child Health Care Hospital, Beijing 100006, China; Reproductive Medicine Center, Affiliated Yantai Yuhuangding Hospital of Qingdao University, Yantai 264000, China; College of Life Sciences, Beijing Normal University, Beijing 100875, China; National Institute of Biological Sciences, Beijing 102206, China; National Institute of Biological Sciences, Beijing 102206, China; Department of Reproductive Medicine, The First Affiliated Hospital of Xiamen University, School of Medicine, Xiamen University, Xiamen 361003, China; National Institute of Biological Sciences, Beijing 102206, China; College of Life Sciences, Beijing Normal University, Beijing 100875, China; National Institute of Biological Sciences, Beijing 102206, China; Reproductive Medicine Center, Affiliated Yantai Yuhuangding Hospital of Qingdao University, Yantai 264000, China; National Institute of Biological Sciences, Beijing 102206, China; Academy for Advanced Interdisciplinary Studies (AAIS), and Peking University–Tsinghua University—National Institute of Biological Sciences Joint Graduate Program (PTN), Peking University, Beijing 100871, China; National Institute of Biological Sciences, Beijing 102206, China; Department of Reproductive Medicine, Quanzhou Women and Children’s Hospital, Quanzhou 362000, China; National Institute of Biological Sciences, Beijing 102206, China; National Institute of Biological Sciences, Beijing 102206, China; National Institute of Biological Sciences, Beijing 102206, China; Academy for Advanced Interdisciplinary Studies (AAIS), and Peking University–Tsinghua University—National Institute of Biological Sciences Joint Graduate Program (PTN), Peking University, Beijing 100871, China; Central Laboratory, Beijing Obstetrics and Gynecology Hospital, Capital Medical University, Beijing Maternal and Child Health Care Hospital, Beijing 100006, China; National Institute of Biological Sciences, Beijing 102206, China; Tsinghua Institute of Multidisciplinary Biomedical Research, Tsinghua University, Beijing 100084, China

**Dear Editor,**

Fertilization is crucial for the beginning of the life cycle of humans and other mammals. Capacitated spermatozoa recognize, interact, and fuse with oocytes, and the zygote develops into an embryo. Both female and male factors can cause fertilization failure. The genetic pathogenic factors of more than half of men with fertilization failure have not been identified [1, 2]. Therefore, it is important to determine the molecular cause of fertilization failure. In this study, we first identified an infertile patient with fertilization failure from a consanguineous family (Family 1; Figs. 1A and S1A). During *in vitro* fertilization (IVF), the sperm from the patient could not fertilize the oocytes from his wife (Table S1). Following intracytoplasmic sperm injection (ICSI), most zygotes were unable to divide and arrested at the one-cell stage (Fig. S1A; Table S1). With the patient’s consent, whole-exome sequencing (WES) was performed on the infertile patient. Variants with allele frequencies in databases [e.g., Genome Aggregation Database (gnomAD), Exome Aggregation Consortium (ExAC), and 1000 Genomes (1000G)] >1% were filtered out, and the retained rare homozygous sequence variants were analyzed. Among the retained genes, *IQCN* was the only gene that was specifically and highly expressed in the human testis (the Human Protein Atlas website). A rare truncating homozygous variant, c.2453_2454del (p.Q818Rfs*9), was identified in *IQCN*. Subsequently, two infertile patients from two independent families with fertilization failure and zygotic arrest were recruited for this study (Table S1). The wife of the patient in Family 2 had a history of pregnancy with her ex-boyfriend, thereby indicating that the male factor may be the predominant cause of fertilization failure and zygotic arrest. WES also identified that II-1 in Family 2 had the same truncating homozygous variant, c.2453_2454del (p.Q818Rfs*9) in *IQCN* as II-1 in Family 1 (Fig. 1A). II-1 in Family 3 had the compound heterozygous variants c.3008C>T (p.S1003F) and c.3374G>A (p.R1125H) in *IQCN* (Fig. 1A). Sanger sequencing validated all the variants in each infertile patient and suggested an autosomal recessive mode of inheritance (Fig. 1A). The allele frequencies of these variants in human populations are very low, and they were absent from the gnomAD database (Table S2). The amino acid sites of two missense variants, p.S1003F and p.R1125H, were evolutionarily conserved (Fig. S1B). The c.2453_2454del variant was predicted to produce a truncated p.Q818Rfs*9 protein, which would destroy the five IQ1 domains of the IQCN protein and lead to the loss of the C-terminal sequences (Fig. S1B). These findings indicate a possible genetic contribution of *IQCN* to fertilization failure, male infertility, and early embryonic arrest, including zygotic arrest.

**Figure 1.**
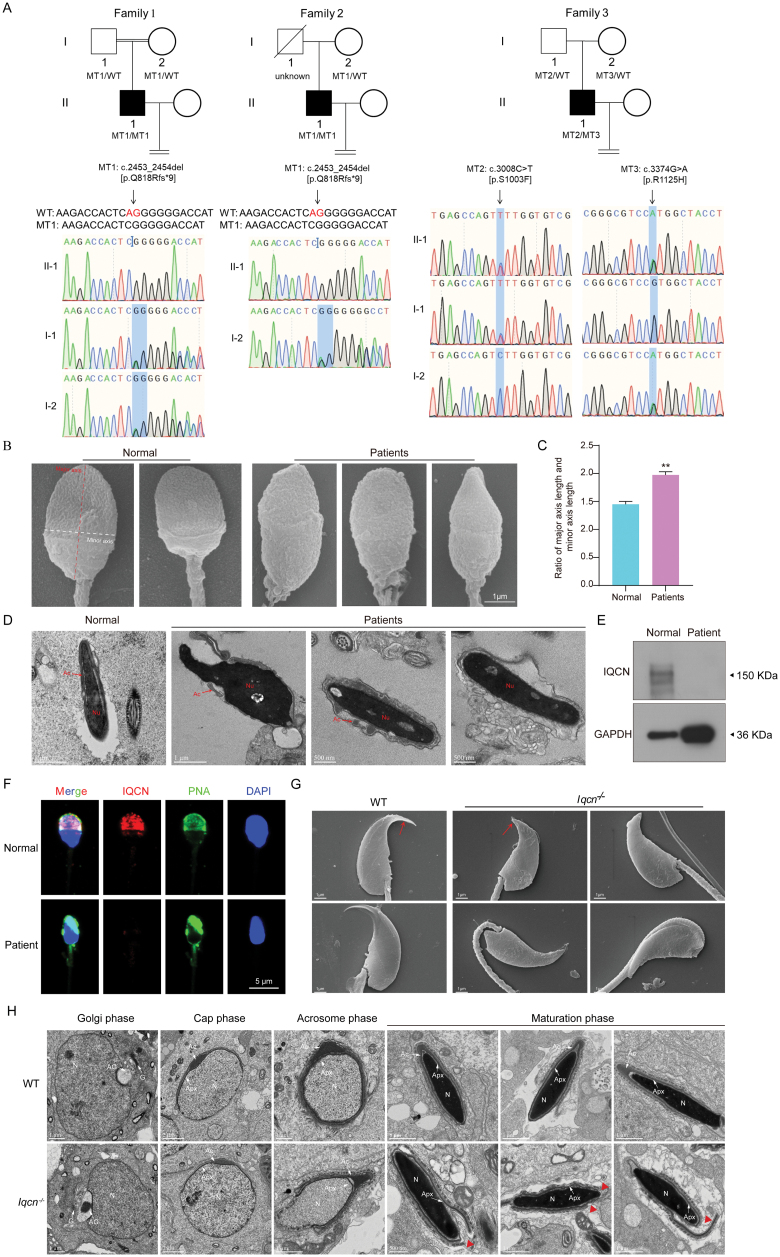
*IQCN/Iqcn* deficiency cause fertilization failure and sperm acrosomal defects in humans and mice. (A) Pedigrees and Sanger sequencing validation of *IQCN* in three patients with fertilization failure. Filled black squares indicate affected individuals. Black arrows indicate the mutational sites. The red AG nucleotides are deleted in the homozygous state in the affected individuals in Family 1 and Family 2, respectively. WT, wild type; MT, mutant. (B) Ultrastructure of spermatozoa determined by SEM from a fertile normal control and patients with biallelic *IQCN* variants. Spermatozoa from men with biallelic *IQCN* variants display tapered sperm heads with longer major axis and shorter minor axis. Red line indicates the major axis and the white dotted line indicates the minor axis. Scale bars: 1 μm. (C) The ratio of major to minor axis length in spermatozoa from a fertile normal control and patients with biallelic *IQCN* variants. 30 sperms were quantified. (D) Ultrastructure of spermatozoa determined by TEM from a fertile normal control and patients with biallelic *IQCN* variants. In a fertile normal control, the red arrow indicates the normal acrosome in spermatozoa. In the spermatozoa from patients with biallelic *IQCN* variants, the red arrows indicate the fragmented acrosomes. In the right image, no acrosomes are found in the spermatozoa. Nu, nucleus; Ac, acrosome. Scale bars: 500 nm and 1 μm. (E) Western blot analysis of IQCN protein expression in the patient. The patient is harboring the IQCN compound heterozygous variants p.S1003F and p.R1125H. GAPDH protein is as loading control. (F) Immunofluorescence staining of IQCN in ejaculated sperm from a fertile normal control and patient with biallelic *IQCN* variants. Anti-IQCN (red) antibody and peanut agglutinin (PNA, green) were used. The nuclei of sperm are 4ʹ,6-diamidino-2-phenylindole (DAPI) labeled (blue). Scale bars: 5 μm. (G) Ultrastructure of spermatozoa determined by SEM from WT and *Iqcn*^−/−^ male mice. Acrosomal morphology of spermatozoa from WT mice is sickle shape, whereas the hook rim of the spermatozoa from *Iqcn*^−/−^ male mice is shorter and lacks a sharp tip, and the heads of some *Iqcn*^−/−^ sperm are twisted toward the flagella. The red arrows indicate the hook rims of sperm. Scale bars: 1 μm. (H) Acrosome biogenesis of spermatids from WT and *Iqcn*^−/−^ male mice. Four phases of acrosome biogenesis are included: Golgi, cap, acrosomal, and maturation. N, nucleus; AG, acrosomal granule; G, Golgi; Ac, acrosome; Apx, acroplaxome. Scale bars: 500 nm, 1 μm, and 2 μm.

Three *IQCN*-mutated individuals exhibited normal sperm parameters (Table S3). To study sperm morphology, hematoxylin-eosin (HE) staining, scanning electron microscopy (SEM), and transmission electron microscopy (TEM) were performed. The percentage of normal sperm in all three individuals was above the reference limit (4%) (Table S3). However, the three individuals had an increased proportion of tapered-head sperm, which showed an increased ratio of major to minor axis length (Figs. 1B, 1C and S1C). Using TEM, compared with normal sperm, most acrosomes of mutant sperm were detached from the nuclear envelope and were missing (Fig. 1D). A few mutant sperms had acrosomes, however, the morphology of such acrosomes was abnormal and showed fragmentation (Fig. 1D). The sperm acroplaxome of the *IQCN*-mutated patient was also detached from the nuclear envelope and showed shedding and folding (Fig. 1D). Western blot analysis was also performed using the sperm sample from the patient harboring p.S1003F and p.R1125H compound heterozygous variant, and we found that IQCN protein was failed to be detected in the patient (Fig. 1E). Immunofluorescence staining showed that the IQCN protein was expressed in the acrosome of normal sperm, however, it was barely expressed in the mutant sperm (Fig. 1F). ACRV1, an acrosomal matrix protein, was expressed well in the acrosome of normal sperm, however, it was expressed as fragments in the head of mutant sperm and was mis-localized in the flagella of sperm (Fig. S1D), indicating that the integrity of the acrosome was destroyed in mutant sperm. PLCZ1 expression was abnormal characterized as irregular scattered points in the sperm head (Fig. S1E). These observations indicate that *IQCN* mutations are associated with acrosomal defects in the human sperm.

Next, we studied the role of Iqcn in mouse genetics. Mouse Iqcn was expressed during spermiogenesis, however, not during the early stage of spermatogenesis (Fig. S2A and S2B), and the expression pattern was very similar to that in humans (Fig. S1C and S1D). To test how *IQCN* mutations affect acrosomal defects, *Iqcn*^−/−^ mice were generated using CRISPR-Cas9 technology (Fig. S3A and S3B). There were no significant differences in testis size or weight between WT and *Iqcn*^−/−^ male mice (Fig. S3C and S3D). HE staining of the testis, caput, and cauda showed no significant differences in spermatogenesis between WT and *Iqcn*^−/−^ male mice (Fig. S3E). Periodic acid–Schiff staining of different steps of spermiogenesis revealed no significant differences between WT and *Iqcn*^−/−^ male mice (Fig. S3F). To test the fertility of *Iqcn*^−/−^ mice, male *Iqcn*^−/−^ mice were mated with WT mice. We found that *Iqcn*^−/−^ mice were infertile (Table S4). Coomassie staining showed that acrosomal morphology changed from a sickle shape in WT mice to a comma or dot shape in *Iqcn*^−/−^ mice, and the heads of some *Iqcn*^−/−^ sperm turned in the direction of the flagella (Fig. S4A). The above findings were also confirmed by SEM, which showed that the hook rim of spermatozoa from *Iqcn*^−/−^ mice was shorter and lacked a sharp tip (Fig. 1G). TEM showed that the acrosome was detached from the nuclear envelope in the ejaculated sperm of *Iqcn*^−/−^ mice, consistent with that of the sperm from the human patients (Fig. S4B). Peanut agglutinin (PNA) staining of the ejaculated sperm from *Iqcn*^−/−^ male mice also showed an acrosomal defect phenotype (Fig. S4C). Many acrosomes shed from the sperm head were found in the epididymis (Fig. S4D). Immunofluorescence staining showed that the Iqcn protein was expressed in the acrosome of WT mice although barely expressed in *Iqcn*^−/−^ mice (Fig. S4E). Plcz1 was expressed in the perinuclear theca, a subacrosomal region in the sperm of WT mice; however, in sperm from *Iqcn*^−/−^ mice, Plcz1 was not expressed under the acrosome and was instead expressed as irregular scattered points in the sperm head (Fig. S4F). These findings showed that the phenotypes of *Iqcn*^−/−^ mice were identical to those of human patients harboring *IQCN* variants. These data confirm that *IQCN* deficiency causes male infertility through sperm acrosomal defects.

Next, we investigated the mechanism by which *IQCN* deficiency causes acrosomal defects in sperm. We examined the structural details of spermiogenesis in WT and *Iqcn*^−/−^ mice using TEM. During the cap phase, the acrosomal cap was normally formed in WT and *Iqcn*^−/−^ mice (Fig. 1H). In the acrosome phase, no significant defects were found in *Iqcn*^−/−^ mice (Fig. 1H). However, the inner membrane of the acrosome detached from the nuclear envelope during the maturation phase (Fig. 1H). These observations suggest that the integrity of the acrosome appears to be intact during the cap phase. However, the integrity of the acroplaxome, which anchors the acrosome to the nuclear envelope, may be destroyed during the maturation phase.

To further study the function of Iqcn in sperm head development, two lines (7# and 13#) of transgenic mice expressing *Iqcn-3×FLAG* were successfully generated (Fig. S5A–S5C). IF staining analysis of ejaculated sperm from *Iqcn-3×FLAG*^+/+^ mice revealed that Iqcn was expressed along the acroplaxome and predominantly in the marginal ring region of the acroplaxome (Fig. S6A). Iqcn was also co-localized with Acrv1 and Actl7a along the acroplaxome (Fig. S6B and S6C). To examine the *Iqcn* expression pattern, IF staining was performed on testis sections, and we found that *Iqcn* was first expressed around step 11 of spermiogenesis (Fig. S6D). We also isolated spermatids from the testes and performed IF staining, which clearly showed that *Iqcn* expression began at step 8 and was localized in the subacrosomal layer, otherwise known as the acroplaxome (Fig. S6E). In the above two experiments, the different expression start times of Iqcn may be caused by incomplete antigen repair during the experiment in the testis section. Actl7a is an acroplaxome marker [3]; and we observed that Iqcn co-localized with Actl7a in the acroplaxome during spermatid elongation (Fig. S7A and S7B). Actb also localized along the acroplaxome [4]. Our study also found that Iqcn co-localized with Actb during spermiogenesis (Fig. S8A and S8B). Therefore, the staining results of Iqcn in sperm suggests that *Iqcn* deficiency may cause abnormal formation of the acroplaxome, resulting in detachment of the acrosome from the nuclear envelope.

To elucidate the molecular mechanism of Iqcn during spermatid elongation, we identified Iqcn interacting proteins by mass spectrometry (Fig. S9A). Among these interacting proteins, Actb, Actl7a, and Actbl2 were carefully examined. We first examined Actb, Actl7a, and Actbl2 localization in patients harboring *IQCN* mutations and in *Iqcn*^−/−^ male mice. In the sperm head from the normal control, ACTB, ACTL7A, and ACTBL2 were mainly localized in the acrosome (Figs. 2A, S9B and S9C), whereas in the sperm from the patients, ACTB and ACTBL2 expression changed from normal scattered expression to condensed dotted expression (Figs. 2A and S9C), and ACTL7A expression was also abnormal (Fig. S9B). Similar results were found in sperm from *Iqcn*^−/−^ male mice (Figs. 2B, S9D and S9E), and it is worth noting that Actl7a was also expressed in the marginal ring region of the acroplaxome in the sperm head from WT; however, in sperm from *Iqcn*^−/−^ male mice, the marginal ring region of the acroplaxome failed to form well (Fig. S9D). These observations suggest that the correct localization of Actb, Actl7a, and Actbl2 requires the IQCN protein. Second, IP experiments were conducted using testicular tissue extracts and sperm from *Iqcn-3×FLAG*^+/+^ mice, and we confirmed that Iqcn interacts with Actb, Actl7a, Actbl2, Tubb3, Tuba1c, and Capzb (Figs. 2C and S9F–S9J).

**Figure 2.**
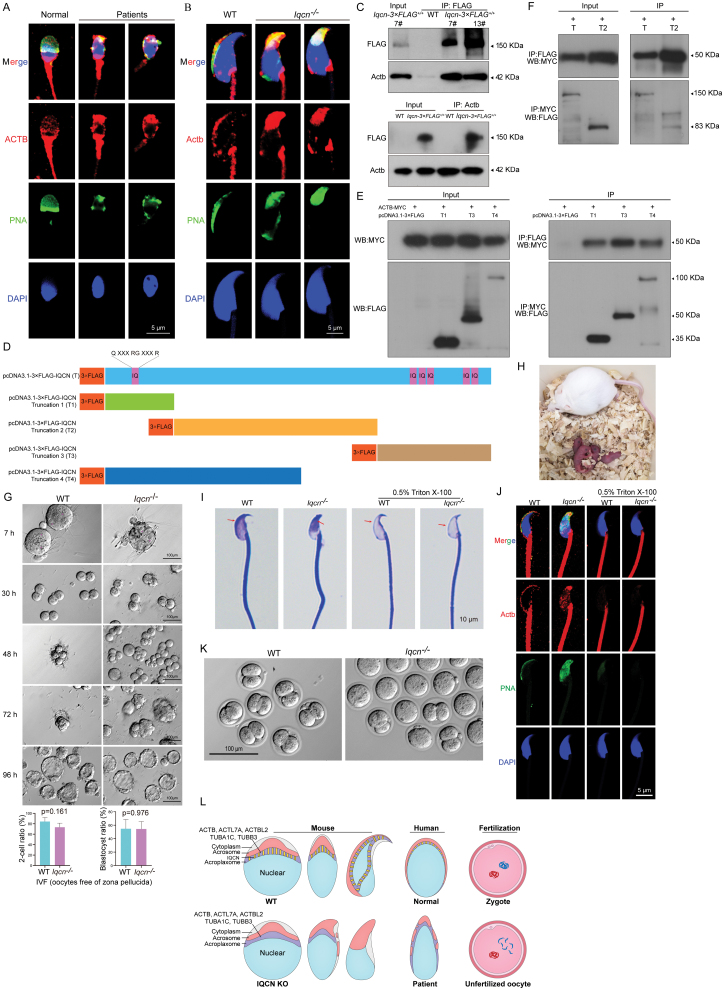
Iqcn maintains acroplaxome integrity by interacting with Actb, *Iqcn*^−/−^ sperm can fertilize the oocytes free of zona pellucida, normal oocytes could be fertilized via ICSI followed by AOA treatment using *Iqcn*^−/−^ sperm without acrosome. (A) Immunofluorescence (IF) staining of ACTB in spermatozoa from a fertile normal control and patients with biallelic *IQCN* variants. Anti-ACTB (red) antibody and PNA (green) were used. The nuclei of sperm are DAPI labeled (blue). Scale bars: 5 μm. (B) IF staining of Actb in spermatozoa from WT and *Iqcn*^−/−^ male mice. Anti-Actb (red) antibody and PNA (green) were used. The nuclei of sperm are DAPI labeled (blue). Scale bars: 5 μm. (C) Interaction between *Iqcn* and Actb following co-immunoprecipitation (co-IP) in the testes of two *Iqcn-3×FLAG*^+/+^ mice (7# and 13#). FLAG-tagged Iqcn interacts with Actb. (D) Diagram of construction plasmids of different IQCN truncations. (E) Interaction between each IQCN truncation (T1, T3, or T4) and ACTB following co-IP in cells transfected with FLAG-tagged IQCN truncations and MYC-tagged ACTB. Each IQCN truncation interacts with ACTB. (F) Interaction between full length IQCN or T2 truncation and ACTB following co-IP in cells transfected with FLAG-tagged IQCN or T2 truncation and MYC-tagged ACTB. Full length IQCN or T2 truncation interacts with ACTB. (G) IVF-enabled sperm from *Iqcn*^−/−^ male mice to fertilize the oocytes free of zona pellucida. There was no difference in two-cell formation rate and blastocyst formation rate between WT and *Iqcn*^−/−^ male mice. Bars indicate means ± standard error of the mean (SEM). 217 and 261 oocytes were used in WT and *Iqcn*^−/−^ group, respectively. (H) Representative image of the offspring of *Iqcn*^−/−^ male mice born after fertilization of oocytes free of zona pellucida. (I) 0.5% Triton X-100 was used to remove the acrosome in spermatozoa from WT and *Iqcn*^−/−^ male mice. The red arrows point to the acrosome region. Scale bars: 10 μm. (J) IF staining of Actb in spermatozoa from WT and *Iqcn*^−/−^ male mice after 0.5% Triton X-100 treatment. Anti-Actb antibody (red) and PNA (green) were used, respectively. The nuclei of sperm are DAPI labeled (blue). Scale bars: 5 μm. (K) *Iqcn*^−/−^ sperm without acrosome could fertilize normal oocytes via ICSI followed by artificial oocyte activation (AOA) treatment. Scale bars: 100 μm. (L) Schematic models for the function of IQCN. Schematic model (upper) showed that IQCN and its interacting proteins (such as ACTB, ACTL7A, ACTBL2) localized in the acroplaxome, and played pivotal roles in sperm head shaping and fertilization in spermatids from human or mice. Schematic model (lower) of abnormal sperm head shaping and acrosome localization observed in *IQCN/Iqcn* deficiency spermatids from human or mice.

To clarify the function of IQCN protein precisely, plasmids containing FLAG-tagged full length human IQCN, N-terminal (T1), middle region (T2), C-terminal (T3), N-terminal plus part of middle region IQCN (T4), MYC-tagged human ACTB have been constructed (Fig. 2D). After co-transfected into cells, we found that the full length, and all of the different truncation proteins (T1, T2, T3, and T4) can interact with ACTB (Fig. 2E and 2F), indicating that IQCN might function as a scaffold protein and interact with actin associated filaments.

Next, we investigated whether IVF/ICSI treatment could overcome the adverse effects of the identified acrosomal defects. IVF using the sperm of *Iqcn*^−/−^ mice failed to fertilize normal oocytes and oocytes free of cumulus cells from WT mice (Fig. S10A). After ICSI, none of the oocytes were successfully fertilized (Fig. S10A), which was potentially caused by failure to form male pronuclei (Fig. S10B). These results showed that neither IVF nor ICSI could rescue infertility in *Iqcn*^−/−^ mice, similar to the patients exhibiting *IQCN* mutations. We also used the artificial oocyte activation (AOA) method, which has been proposed as a suitable method for restoring fertilization. AOA using strontium chloride (SrCl_2_) or calcium ionophore ionomycin following ICSI was performed, however, neither could successfully divide the zygote (Fig. S10A). Although we occasionally observed embryo division into two cells, subsequent development failed. The above experiment showed that AOA could not rescue zygotic arrest caused by the *Iqcn* defect.

We explored other avenues for resolving infertility. We further removed the zona pellucida of the oocytes and determined whether *Iqcn*^−/−^ sperm could fertilize the oocytes by IVF and subsequently undergo zygotic cleavage. Surprisingly, the *Iqcn*^−/−^ sperm-fertilized oocytes were divided into the two-cell stage and subsequently developed into blastocysts (Fig. 2G). There were no significant differences between the oocytes fertilized by normal sperm and those fertilized by *Iqcn*^−/−^ sperm developing into two cells (87.6% vs. 72.4%, *P* = 0.161) or blastocysts (48.2% vs. 55.6%, *P* = 0.976) (Fig. 2G). To further investigate whether *Iqcn*^−/−^ male mice can produce their own offspring, blastocysts were transplanted into pseudo-pregnant mice. Embryos were successfully implanted and developed into four pups (Fig. 2H), which were validated by genotyping (Fig. S10C). These four pups developed normally to postnatal day 15 (P15) (Fig. S10D). In addition, the experiment was repeated; embryos were also successfully implanted and developed into two pups (Fig. S10E), which were validated by genotyping (Fig. S10E). Further observation showed that the offsprings that harbored one allele *Iqcn* KO (*Iqcn*^+/−^) were fertile, and the subsequent offsprings that harbored biallelic *Iqcn* KO (*Iqcn*^−/−^) were sterile. All these prove that the method of using *Iqcn*^−/−^ sperm to fertilize the oocytes that removal the zona pellucida is effective.

The above findings showed that the sperm of *Iqcn*^−/−^ male mice can normally fertilize oocytes without the zona pellucida, indicating that there is no problem with adhesion and membrane fusion mediated by Izumo-Juno interaction [5], and the sperm borne oocyte-activating factor [6] can be normally released into the oocyte upon gamete fusion. *Iqcn*^−/−^ sperm could not fertilize the oocytes by IVF, which might be due to defects in the acrosome reaction. To assess the capacitation and acrosome reaction, Coomassie brilliant blue staining of sperm was performed: staining of acrosomes disappeared after acrosome reaction in sperm from WT mice (Fig. S11A); however, staining did not completely disappear in sperm from *Iqcn*^−/−^ male mice (Fig. S11A), suggesting a defective acrosome reaction in sperm from *Iqcn*^−/−^ male mice.

*Iqcn*^−/−^ sperm could not fertilize the oocytes by ICSI or ICSI followed by AOA treatment. The most likely reason is that the acrosome of the *Iqcn*^−/−^ sperm contains factors that inhibit both the loosening of sperm chromatin and activation of oocytes. Therefore, we removed the acrosome of the *Iqcn*^−/−^ sperm and performed ICSI followed by AOA treatment to confirm whether this variation could fertilize normal oocytes. After 0.5% Triton X-100 treatment, sperm from both WT and *Iqcn*^−/−^ male mice lost the acrosome (Fig. 2I). Immunostaining of the acrosomal proteins, including Actb, Actl7a, Actbl2, Acrv1, Plcz1, and Tubb3 in treated sperm suggested that the acrosome was removed (Figs. 2J and S11B–S11F). *Iqcn*^−/−^ male pronuclei could not be formed using the traditional ICSI treatment (Fig. S12A). However, after 0.5% Triton X-100 treatment, *Iqcn*^−/−^ male pronuclei formed (Fig. S12B). Furthermore, the *Iqcn*^−/−^ sperm without acrosome-derived zygotes can develop to the two-cell stage even if the efficiency is relatively low (Fig. 2K). Eventually, one pup was obtained (Fig. S12C).

In conclusion, we have identified a novel gene that causes male infertility due to fertilization failure. Genetic and experimental evidence from *IQCN*-associated men and *Iqcn*^−/−^ male mice strongly supports that IQCN plays a vital role in anchoring the inner acrosomal membrane to the nuclear envelope. IQCN deficiency causes the acrosome to detach from the nuclear envelope, leading to fertilization failure (Fig. 2L). Our study provides new molecular insights into acrosomal biogenesis and fertilization.

## Research limitations

This study has one major limitation that should be addressed in future research. Although we found that the infertile *Iqcn*^*−/−*^ sperm could fertilize oocytes without zona pellucida in mice and obtain healthy offspring, we did not use this method with the male patients in clinical practice, due to the lack of treatment guidelines for this issue in China, and the restrictions on ethical responsibility in the removal of zona pellucida from oocytes. It has been reported that zona pellucida manipulation might improve fertilization in severe male factor infertility [7], decrease cytoplasmic fragmentation in human embryos [8], and enhance the rate of embryonic implantation and pregnancy [9], without affecting the tested parameters of clinical outcomes [10]. This evidence could persuade patients to undergo similar therapeutic regimens in clinical practice.

## Supplementary Material

lnac050_suppl_Supplementary_Material

## References

[1] Dai J, Zhang T, Guo J, et al. Homozygous pathogenic variants in ACTL9 cause fertilization failure and male infertility in humans and mice. Am J Hum Genet 2021;108:469–81. 33626338 10.1016/j.ajhg.2021.02.004 PMC8008497

[2] Xin A, Qu R, Chen G, et al. Disruption in ACTL7A causes acrosomal ultrastructural defects in human and mouse sperm as a novel male factor inducing early embryonic arrest. Sci Adv 2020;6:eaaz4796. 32923619 10.1126/sciadv.aaz4796 PMC7455188

[3] Boëda B, Knowles PP, Briggs DC, et al. Molecular recognition of the Tes LIM2-3 domains by the actin-related protein Arp7A. J Biol Chem 2011;286:11543–54. 21278383 10.1074/jbc.M110.171264 PMC3064208

[4] Kierszenbaum AL, Rivkin E, Tres LL. Acroplaxome, an F-actin-keratin-containing plate, anchors the acrosome to the nucleus during shaping of the spermatid head. Mol Biol Cell 2003;14:4628–40. 14551252 10.1091/mbc.E03-04-0226 PMC266778

[5] Bianchi E, Doe B, Goulding D, et al. Juno is the egg Izumo receptor and is essential for mammalian fertilization. Nature 2014;508:483–7. 24739963 10.1038/nature13203 PMC3998876

[6] Kimura Y, Yanagimachi R, Kuretake S, et al. Analysis of mouse oocyte activation suggests the involvement of sperm perinuclear material. Biol Reprod 1998;58:1407–15. 9623599 10.1095/biolreprod58.6.1407

[7] Odawara Y, Matsumoto K, Shinozuka S, et al. Zona manipulation in an assisted reproductive technologies (ART) programme. J Obstet Gynaecol (Tokyo 1995) 1995;21:83–8. 8591115 10.1111/j.1447-0756.1995.tb00902.x

[8] Yumoto K, Shimura T, Mio Y. Removing the zona pellucida can decrease cytoplasmic fragmentations in human embryos: a pilot study using 3PN embryos and time-lapse cinematography. J Assist Reprod Genet 2020;37:1349–54. 32285294 10.1007/s10815-020-01773-y PMC7311590

[9] Mansour RT, Rhodes CA, Aboulghar MA, et al. Transfer of zona-free embryos improves outcome in poor prognosis patients: a prospective randomized controlled study. Hum Reprod 2000;15:1061–4. 10783352 10.1093/humrep/15.5.1061

[10] Kirienko KV, Apryshko VP, Naumova AA, et al. Mechanical zona pellucida removal of vitrified-warmed human blastocysts does not affect the clinical outcome. Reprod Biomed Online 2019;39:745–9. 31530444 10.1016/j.rbmo.2019.06.003

