# Supplementary material for: Biallelic mutations in IQCN, encoding a novel acroplaxome protein, lead to fertilization failure and male infertility with defects in the acrosome and shaping of the spermatid head in humans and mice
Source: Life Med. 2022 Nov 4;2(2):lnac050. doi: 10.1093/lifemedi/lnac050 (PMC11749140; doi:10.1093/lifemedi/lnac050)
Supplement: lnac050_suppl_Supplementary_Material [file lnac050_suppl_Supplementary_Material.docx]

**Supplementary Information for “Biallelic mutations in *IQCN*, encoding a novel acroplaxome protein, lead to fertilization failure and male infertility with defects in the acrosome and shaping of the spermatid head in humans and mice”**

**Materials and Methods**

**Study Subjects**

Two infertile men with fertilization failure were recruited from the Department of Andrology, Women and Children’s Hospital, School of Medicine, Xiamen University (Xiamen, Fujian, China). One infertile man with fertilization failure was recruited from the Reproductive Medicine Center, Affiliated Yantai Yuhuangding Hospital of Qingdao University (Yantai, Shandong, China). The karyotype of each patient was normal 46, XY, with no microdeletions on the Y chromosome. Physical examination of each patient showed normal development of the male external genitalia, normal bilateral testis size, and no abnormalities in the bilateral spermatic veins upon palpation.

This study was approved by the Ethics Committees of the Women and Children’s Hospital, School of Medicine, Xiamen University (KY-2019-060) and the Medical College of Qingdao University (QDU-HEC-2022109). All individuals recruited for this study provided written informed consent.

**WES and *In-silico* Bioinformatic Analysis**

Genomic DNA was extracted from whole peripheral blood samples from each patient using the QIAamp DNA Blood Kit (Qiagen, Valencia, CA, USA) according to the manufacturer’s protocol. WES was performed as previously described ([Sha et al., 2021](#_ENREF_4)). Sanger sequencing was performed to validate the sequence variants in each patient. The primers used for polymerase chain reaction (PCR) and sequencing are listed in Table S5.

**Animals**

The mice were housed at the National Institute of Biological Sciences according to the Ministry of Health National Guidelines for Housing and Care of Laboratory Animals. All the experiments were approved by the Institutional Animal Care and Use Committee of the National Institute of Biological Sciences.

**Mouse Model Generation Using CRISPR/Cas9**

*Iqcn* knockout (KO) (*Iqcn*^‒/‒^) and *Iqcn*-*3×FLAG*^+/+^ mice were generated by co-microinjection of *in vitro*-translated Cas9 protein and guide RNA (gRNA) into C57BL/6 zygotes. C57BL/6 wild-type (WT) mice were obtained from Beijing Vital River Laboratory Animal Technology Co., Ltd. The gRNA sequences used to generate *Iqcn*^‒/‒^ mice were ccccggaggtggtctccatccac and gggagtctgtcggtggcgtgggg. The gRNA sequence used to generate *Iqcn*-*3×FLAG*^+/+^ mice was cctggcgtctagacccctccctg.

**Human and Mouse Semen Parameter Analysis**

Routine semen analysis was conducted for each patient, according to the guidelines of the World Health Organization Laboratory Manual for the Examination and Processing of Human Semen (fifth edition). Semen volume, sperm concentration, total motility, and the percentage of normal spermatozoa were analyzed during routine examinations. Hematoxylin-eosin (HE) staining was performed to assess spermatozoa morphology.

Sperm were obtained from the caudal epididymis of wild-type (WT) and *Iqcn*^-/-^ male mice (10-12 weeks). Each caudal epididymis was placed in 300 μL of human tubal fluid (HTF) medium for capacitation for 30 min at 37 °C. Sperm morphology was analyzed by fixing in 2% paraformaldehyde (PFA) and staining with G250 for five to 10 min, followed by washing and microscopic examination. Semen characteristics were analyzed using a computer-assisted sperm analysis (CASA) system (Version.12 CEROS, Hamilton Thorne Research, USA).

The acrosome reaction was assessed as previously discussed ([Chen et al., 2022](#_ENREF_1)), WT and *Iqcn*^-/-^ spermatozoa were exposed to 10 μM calcium ionophore (A23187, 21186-5MG-F; Sigma-Aldrich, MO, USA) for 30 min to induce the acrosome reaction, and the control group was exposed to DMSO. Sperm were fixed in 2% PFA and spotted onto glass slides for staining with G250 for five to 10 min, followed by washing and microscopic examination.

**Scanning Electron Microscopy (SEM) and Transmission Electron Microscopy (TEM)**

Human and mouse semen samples were prepared for SEM and TEM as described previously ([Chen et al., 2022](#_ENREF_1)). For the SEM assay, spermatozoa were fixed in 4% PFA overnight at 4°C. The specimens were then deposited on poly L-lysine-coated coverslips and air dried. Subsequently, the coverslips were dehydrated using an ascending gradient of ethanol concentration at room temperature, coated with gold particles, and observed using an EVO LS10 SEM (Carl Zeiss NTS, Germany).

For TEM, samples including testis, seminiferous tubules, and sperm cells were fixed with 2.5% glutaraldehyde (G5882, Sigma-Aldrich, USA), which was followed by treatment with 1% osmium tetraoxide for 1 h and with 2% uranyl acetate overnight at 4°C. Subsequently, dehydration was performed with a series of graded acetone solutions using the progressive lowering temperature method. The samples were infiltrated and embedded in Epon 812. Ultrathin (90 nm in thickness) sections were stained with 3% uranyl acetate and lead citrate prior to TEM using a Tecnai G2 Spirit (Philips/FEI, USA) instrument with an acceleration voltage of 120 kV.

**Isolation of Testicular Elongating Spermatids**

Isolation of different types of spermatids from the testes was performed using standard protocols as previously described ([Da Ros et al., 2019](#_ENREF_2)). Briefly, WT and *Iqcn*-*3×FLAG* testes were collected from two-month-old mice, and sequentially digested with collagenase IV and trypsin. Next, the different germ cell types were isolated using a discontinuous bovine serum albumin density gradient. Pachytene spermatocytes, round spermatids, and elongating spermatids were collected and fixed in 4% PFA for immunofluorescence analysis.

**Morphology and Immunofluorescence** **Analysis**

Mouse testes and epididymides were fixed in 4% PFA overnight, sequentially dehydrated with graded ethanol, embedded in paraffin, and cut into 5 μm sections. Human and mouse sperm samples were spread on glass slides and fixed in 4% PFA for 15 min. For morphological analysis, testis sections or sperm smears were stained with HE or G250 ([Chen et al., 2022](#_ENREF_1)). Images were captured using a microscope (VS120; Olympus, Japan). For immunofluorescence, testis sections or spermatid smears were stained with primary antibodies specific to FLAG (#F1804, 1:200; Sigma-Aldrich, USA), Actb (#PM053, 1:200; MBL, Japan), Actl7a (#17355-1-AP, 1:200; Proteintech, China), Actbl2 (#PA5-102030, 1:200; Thermo Fisher Scientific, USA), Tubb3 (#66375-1-Ig, 1:200; Proteintech, China), Tuba1c (#ab222849, 1:200; Abcam, USA), Capzb (25043-1-AP, 1:200; Proteintech, China) and PLCZ1 (#bs-5378R, 1:200; BOSS, China). The sections were stained with Cy3- or Cy5-labeled secondary antibodies (Jackson ImmunoResearch Laboratories, USA) and peanut agglutinin (PNA, #L738, 1:1000; Sigma-Aldrich, USA) before counterstaining with DAPI (#D1306, 1:1000; Invitrogen, USA). Zygotes were stained with primary antibodies specific to α-tubulin (T6199, Sigma-Aldrich, USA). Immunofluorescence images were captured using a Nikon A1 laser scanning confocal microscope (Nikon; N-SIM E, Japan).

**Fertility assessment**

Fertility was assessed using 2:1 caging of WT females with *Iqcn*^‒/‒^ and WT male mice (*n* = 6) for over three months. Successful mating was verified by the presence of a post-coital vaginal plug, after which mice were caged separately, and two-cell stage embryos or pups were analyzed.

***In Vitro* Fertilization and Intracytoplasmic Sperm Injection in Mice**

IVF and ICSI were performed using previously described protocols ([Chen et al., 2022](#_ENREF_1)). Briefly, sperm were isolated from the caudal epididymides (*n* = 3) of eight- to 10-week-old *Iqcn*^‒/‒^ and WT mice. Oocytes were harvested from six-week-old female mice (C57BL/6J background) after the injection of pregnant mare serum gonadotropin and human chorionic gonadotropin (Ningbo Sansheng Biological Technology Co., Ltd., Ningbo, China). The cumulus cells and zona pellucida were removed from the oocytes by treatment with hyaluronidase (H3757, Sigma-Aldrich, USA) and Acidic Tyrode’s Solution (MR-004, Sigma-Aldrich, USA), respectively. The different types of oocytes were incubated with *Iqcn*^‒/‒^ and WT sperm for 4–6 h at 37°C and 5% CO_2_. The number of two-cell embryos at the two-cell or blastocyst stage was counted.

Oocytes free of cumulus cells were transferred to KSOM medium (MR-020P, Sigma-Aldrich, USA) and cultured at 37°C and 5% CO_2_ for ICSI. Single sperm heads from *Iqcn*^‒/‒^ and WT mice were injected into the oocytes of WT mice using a micromanipulator with a piezoelectric actuating pipette. The numbers of two-cell embryos and blastocysts were counted.

**Artificial Oocyte Activation**

AOA was conducted using strontium dichloride (SrCl_2_; 439665, Sigma-Aldrich, USA) and calcium ionophore ionomycin (IONO, 407950, Sigma-Aldrich, USA). Oocytes were exposed to 10 mM SrCl_2_ in KOSM medium for 4 h and 10 μM ionomycin in KOSM medium for 10 min after ICSI. The AOA oocytes were subsequently washed three times in KSOM and incubated for 96 h at 37°C and 5% CO_2_. The number of two-cell embryos was then counted.

**Preparation of Acrosome Free Spermatozoa**

Triton X-100 (Sigma-Aldrich, USA) was used to remove the acrosome inside the *Iqcn*^-/-^ sperm head ([Morozumi et al., 2006](#_ENREF_3)). Briefly, WT and *Iqcn*^-/-^ mouse cauda epididymides were pierced and incubated in 300 μL HTF medium at 37°C and 5% CO_2_ for 30 min to allow spermatozoa to swim up into the medium. The supernatant was centrifuged at 2000 *g* for 3 min at room temperature and washed in PBS three times. The sperm was resuspended in 200 μL of HCZB medium, mixed with an equal volume of HCZB containing 1.0% (v/v) Triton X-100 for 5 min, and washed in HCZB three times by centrifugation (2000 *g* for 3 min) before ICSI. Spermatozoa were fixed in 2% PFA for immunofluorescence (IF).

**Plasmids construction and transfection**

cDNAs encoding Human IQCN, and different IQCN truncations were subcloned into the pcDNA3.1-N-3×FLAG plasmid with a FLAG tag at the N-terminus. cDNAs encoding human ACTB were subcloned into the pcDNA3.1/Myc-His B plasmid with a MYC tag at the C-terminal. The primer sequences for plasmid construction are listed in Table S6.

Transient transfection was performed in 12-well plates using LipoGeneTM 2000 Plus Transfection Reagent (BN17003, Biorigin, Beijing, China) following the manufacturer’s instructions. pcDNA3.1-N-3×FLAG, pcDNA3.1-3×FLAG-IQCN (T), pcDNA3.1-3×FLAG-IQCN N-terminal truncation1 (T1), pcDNA3.1-3×FLAG-IQCN middle (T2), pcDNA3.1-3×FLAG-IQCN C-terminal truncation 3 (T3), and pcDNA3.1-3×FLAG-IQCN truncation 4 (T4) were co-transfected with MYC-tagged human ACTB in HEK293T cells for 48 h. The cell lysis solution was used to perform co-immunoprecipitation.

**Co-immunoprecipitation**

Co-immunoprecipitation was performed using standard protocols as previously described ([Chen et al., 2022](#_ENREF_1)). *Iqcn*-*3×FLAG*^+/+^ and WT testes and sperm were lysed in IP lysis buffer containing 20 mM Tris-HCl (pH 7.6), 150 mM NaCl, 2 mM EDTA, 0.5% np-40, 5% glycerol, and a protease inhibitor cocktail (4693124001, Roche, Germany) for 30 min on ice then centrifuged at 14000 rpm for 20 min at 4°C. Soluble fractions were incubated with anti-FLAG (1:100), Actb (1:100), Actl7a (1:100), Actbl2 (1:100), Tubb3 (1:100), Tuba1c (1:100), and Capzb (1:100) antibodies overnight at 4°C, and then incubated with Dynabeads Protein G (10004D, Invitrogen, USA) or Dynabeads Protein A (10002D, Invitrogen, USA) for 4 h at 4°C with constant rotation. The precipitates were washed six times with IP buffer and the immune complexes were denatured using SDS loading buffer and boiled for 10 min. Thereafter, the samples were loaded onto a 4%–20% SDS-PAGE gel, followed by Western blotting.

**Western Blotting**

Protein samples were lysed in radioimmunoprecipitation assay buffer (50 mM Tris-HCl (pH 7.4), 150 mM NaCl, 1% NP-40, 1 mM EDTA, and 0.1% sodium dodecyl sulfate) containing a protease inhibitor cocktail. Each sample was kept on ice for 30 min and centrifuged at 14000 rpm for 20 min at 4°C. QuickStart Bradford protein 1× dye (#500-0205; Bio-Rad, USA) was used to determine the protein concentration. Proteins were resolved by electrophoresis on precast gels (4–20%) and transferred onto polyvinylidene difluoride membranes (Millipore, Germany). Membranes were blocked with 5% skim milk in Tris-buffered saline containing 0.1% Tween 20 for 1 h and then incubated overnight with primary antibodies diluted in antibody diluent (WB500D; NCM, China) at 4°C. Antibodies specific for anti-FLAG (1:2000), Actb (1:2000), and Actl7a (1:1000) were used. The samples were then incubated with appropriate secondary antibodies conjugated to HRP (#AS014 or AS003, 1:10000; ABclonal Technology, USA) for 1 h. The protein bands were detected using an ECL detection reagent.

**Ovulation Stimulation Protocols, ICSI treatment, and AOA**

The ovarian function and reserves of the patients’ partners were confirmed to be satisfactory. A standard long-term protocol or antagonist ovulation induction protocol was adopted, and follicular development was monitored using vaginal B-ultrasound. After the dominant follicles matured, 6000 IU or 8000 IU of human chorionic gonadotropin was injected, and follicular puncture guided by transvaginal ultrasound was performed 36 to 38 h later. The ICSI procedure has been described previously ([Tan et al. , 2022](#_ENREF_5)). Thirty min after ICSI, the injected oocytes were placed in 5 μM Ca^2+^ carrier A23187 (Sigma-Aldrich, USA) in G-1Plus medium (Vitrolife, Sweden). The oocyte samples were placed in an incubator for heat preservation, moisture preservation, and gas balance for 10 min, then washed in culture drops five to six times, transferred to the G-1Plus medium for culture, and examined for fertilization after 16 to 18 h.

**Statistical Analyses**

Statistical analyses were performed using GraphPad Prism, version 8 (GraphPad Software, San Diego, California USA, www.graphpad.com). Data are expressed as mean ± (standard error of the mean) SEM. Statistical analyses of the differences between two groups were performed using the standard Student *t*-test. *p*-values < 0.05 (denoted by * in Figures) and < 0.01 (denoted by ** in Figures) were considered significant.

**Data availability**

The data supporting the findings of this study are available within the article and its supplementary materials.

**References**

Chen Y, Chen X, Zhang H, Sha Y, Meng R, Shao T, Yang X, Jin P, Zhuang Y, Min W *et al.* TBC1D21 is an essential factor for sperm mitochondrial sheath assembly and male fertility. *Biol Reprod* 2022.

Da Ros M, Lehtiniemi T, Olotu O, Meikar O, Kotaja N. Enrichment of Pachytene Spermatocytes and Spermatids from Mouse Testes Using Standard Laboratory Equipment. *J Vis Exp* 2019.

Morozumi K, Shikano T, Miyazaki S, Yanagimachi R. Simultaneous removal of sperm plasma membrane and acrosome before intracytoplasmic sperm injection improves oocyte activation/embryonic development. *Proc Natl Acad Sci U S A* 2006;**103**:17661-17666.

Sha Y, Liu W, Li L, Serafimovski M, Isachenko V, Li Y, Chen J, Zhao B, Wang Y, Wei X. Pathogenic Variants in ACTRT1 Cause Acephalic Spermatozoa Syndrome. *Front Cell Dev Biol* 2021;**9**:676246.

Tan C, Meng L, Lv M, He X, Sha Y, Tang D, Tan Y, Hu T, He W, Tu C *et al.* Bi-allelic variants in DNHD1 cause flagellar axoneme defects and asthenoteratozoospermia in humans and mice. *Am J Hum Genet* 2022;**109**:157-171.

|  | **IVF and R- ICSI cycles** | | | | | | | **ICSI with AOA cycles** | | | | | | |
| --- | --- | --- | --- | --- | --- | --- | --- | --- | --- | --- | --- | --- | --- | --- |
| **Individual** | **Insemination method** | **Total no. of oocytes** | **No. of mature oocytes** | **Total fertilization rate** | **Normal fertilization rate** | **Good quality embryo rate** | **Available embryo rate** | **Insemination method** | **Total no. of oocytes** | **No. of mature oocytes** | **Total fertilization rate** | **Normal fertilization rate** | **Good quality embryo rate** | **Available embryo rate** |
| II-1 in family 1 | IVF+R-ICSI | 11 | 11 | 27.3% (3/11) | 27.3% (3/11) | 33.3% (1/3) | 66.7% (2/3) | ICSI+AOA | 12 | 9 | 88.9% (8/9) | 33.3% (3/9) | 0% (0/3) | 66.7% (2/3) |
|  | ICSI | 8 | 8 | 12.5% (1/8) | 0% (0/8) | -(0/0) | -(0/0) |  |  |  |  |  |  |  |
|  | ICSI with donor sperm | 8 | 7 | 87.5% (7/8) | 75% (6/8) | 33.3% (1/3) | 66.7% (2/3) |  |  |  |  |  |  |  |
| II-1 in family 2 | IVF+R-ICSI | 14 | 11 | 0% (0/11) | 0% (0/11) | -(0/0) | -(0/0) | ICSI+AOA | 15 | 11 | 18.2% (2/11) | 18.2% (2/11) | 0% (0/2) | 50% (1/2) |
| II-1 in family 3 | ICSI | 8 | 8 | 12.5% (1/8) | 0% (0/8) | -(0/0) | -(0/0) |  |  |  |  |  |  |  |

Table S1. Clinical characteristics of IVF and ICSI attempts in affected individuals

R-ICSI, Rescue Intracytoplasmic Sperm Injection.

Table S2. Overview of the *IQCN* pathogenic variants observed in the three families

|  | II-1 in family 1 and 2 | II-1 in family 3 | II-1 in family 3 |
| --- | --- | --- | --- |
| Gene | *IQCN* | *IQCN* | *IQCN* |
| Transcript | NM_001145304.2 | NM_001145304.2 | NM_001145304.2 |
| c.DNA mutation | c.2453_2454del | c.3008C>T | c.3374G>A |
| Protein change | p.Q818Rfs*9 | p.S1003F | p.R1125H |
| Mutation type | Frameshift, homozygous | Missense, heterozygous | Missense, heterozygous |
| **Allele frequencies in human populations** | | | |
| Allele frequency in gnomAD | 0.0003332 | 0.0000161 | 0.0001467 |
| Number of heterozygotes | 0 | 0 | 0 |
| **Functional prediction** | | | |
| SIFT | N/A | Deleterious | Deleterious |
| PolyPhen-2 | N/A | Probably damaging | Probably damaging |
| MutationTaster | Disease causing | Polymorphism | Polymorphism |
| PROVEAN | N/A | Damaging | Neutral |

Table S3. Semen characteristics and sperm morphology of affected individuals

|  | II-1 in family 1 | II-1 in family 2 | II-1 in family 3 | Low reference limit |
| --- | --- | --- | --- | --- |
| Semen parameters | | | | |
| Semen volume (ml) | 2.2 | 3.0 | 2.6 | 1.5 ^a^ |
| Sperm concentration (10^6^/ml) | 66.6 | 20.5 | 57.6 | 15 ^a^ |
| Total motility (%) | 44.2 | 52 | 38.5 | 40 ^a^ |
| Sperm morphology | | | | |
| Normal spermatozoa (%) | 10 | 4.3 | 8 | 4 ^a^ |

^a^ Reference limits according to the WHO standards.

Table S4. Fertility test of WT and *Iqcn^-/-^* male mice

| Treatment | | Sperm genotype | No. of total embryo | 1-cell (Oocyte) | No. of Zygote | No. of 2-cell | No. of Blastocyst |
| --- | --- | --- | --- | --- | --- | --- | --- |
| Normal mating | | WT | 57 | 7 | N/A | 50 | N/A |
|  |  | *Iqcn^-/-^* | 66 | 60 | N/A | 0 | N/A |
| IVF | | WT | 116 | 7 | 109 | 106 | 58 |
|  |  | *Iqcn^-/-^* | 140 | 140 | 0 | 0 | 0 |
| ICSI | Normal | WT | 68 | 0 | 28 | 40 | 34 |
|  |  | *Iqcn^-/-^* | 50 | 49 | 0 | 1 | 0 |
|  | AOA (SrCl_2_) | *Iqcn^-/-^* | 29 | 27 | 0 | 2 | 0 |
|  | AOA (IONO) | *Iqcn^-/-^* | 33 | 33 | 0 | 0 | 0 |
| IVF (oocytes free of cumulus cells) | | WT | 37 | N/A | N/A | 27 | 22 |
|  |  | *Iqcn^-/-^* | 45 | N/A | N/A | 0 | 0 |
| IVF (oocytes free of zona pellucida) | | WT | 217 | N/A | 199 | 190 | 96 |
|  |  | *Iqcn^-/-^* | 261 | N/A | 207 | 189 | 89 |

Table S5. PCR Primers used in Sanger sequencing validation

| **Genes and variants** | **Forward Primer** | **Reversed Primer** |
| --- | --- | --- |
| *IQCN*: c.2453_2454del | TAACAGGGTCCAAGGTGTCC | AACAGAGTGCGGAGATCCTC |
| *IQCN*: c.3008C>T | GGATCTCCGCACTCTGTTGG | CCTTGCTGGTCTGTGGTCTC |
| *IQCN*: c.3374G>A | ACACTCCTCGGTCATTCCTC | GCAGCGATGGTCAGATACTG |

Table S6. Primers for plasmid construction

| **Cloning**  **Vector** | **Primers’ name** | **5' to 3' sequences** |
| --- | --- | --- |
| pcDNA3.1-N-3×FLAG-IQCN | N3FLAG-BamHI-hIQCN-F | TACAAGGACGATGATGATAAAGGATCCatgacccttcaaggcagagct |
|  | hIQCN-R1 | atcgcaggccccgggcatac |
|  | hIQCN-F2 | gcgaccatcacggcaaagaaccg |
|  | hIQCN-R2 | ccaggacctccttggacagggcc |
|  | hIQCN-F3 | ctgggcagctctgaaccaggccc |
|  | hIQCN–XhoI-R | CGGGTTTAAACGGGCCCTCTAGACTCGAGctagatgccaggccaatgcatgt |
| pcDNA3.1-N-3×FLAG-Truncations | hIQCN1-240aa-3FLAG-XhoI-R | GGCCCTCTAGACTCGAGctagtgtggcaggaaggcca |
|  | hIQCN-241-930aa-3FLAG-BamHI-F | TGATGATAAAGGATCCcagacggtcaccatcag |
|  | hIQCN-241-930aa-3FLAG-XhoI-R | GGCCCTCTAGACTCGAGctactggggcagggctttggtga |
|  | hIQCN-931-1368aa-3FLAG-BamHI-F | TGATGATAAAGGATCCagcatgctgagcatgg |
| pcDNA3.1/Myc-His B | hACTB-MYC- HindIII | AGACCCAAGCTGGCTAGTTAAGCTTgccacc atggat gatgatatcg ccgcgc |
|  | hACTB-MYC-BamHI | ACCACACTGGACTAGTGGATCCgaagcatttgcggtggacgat |


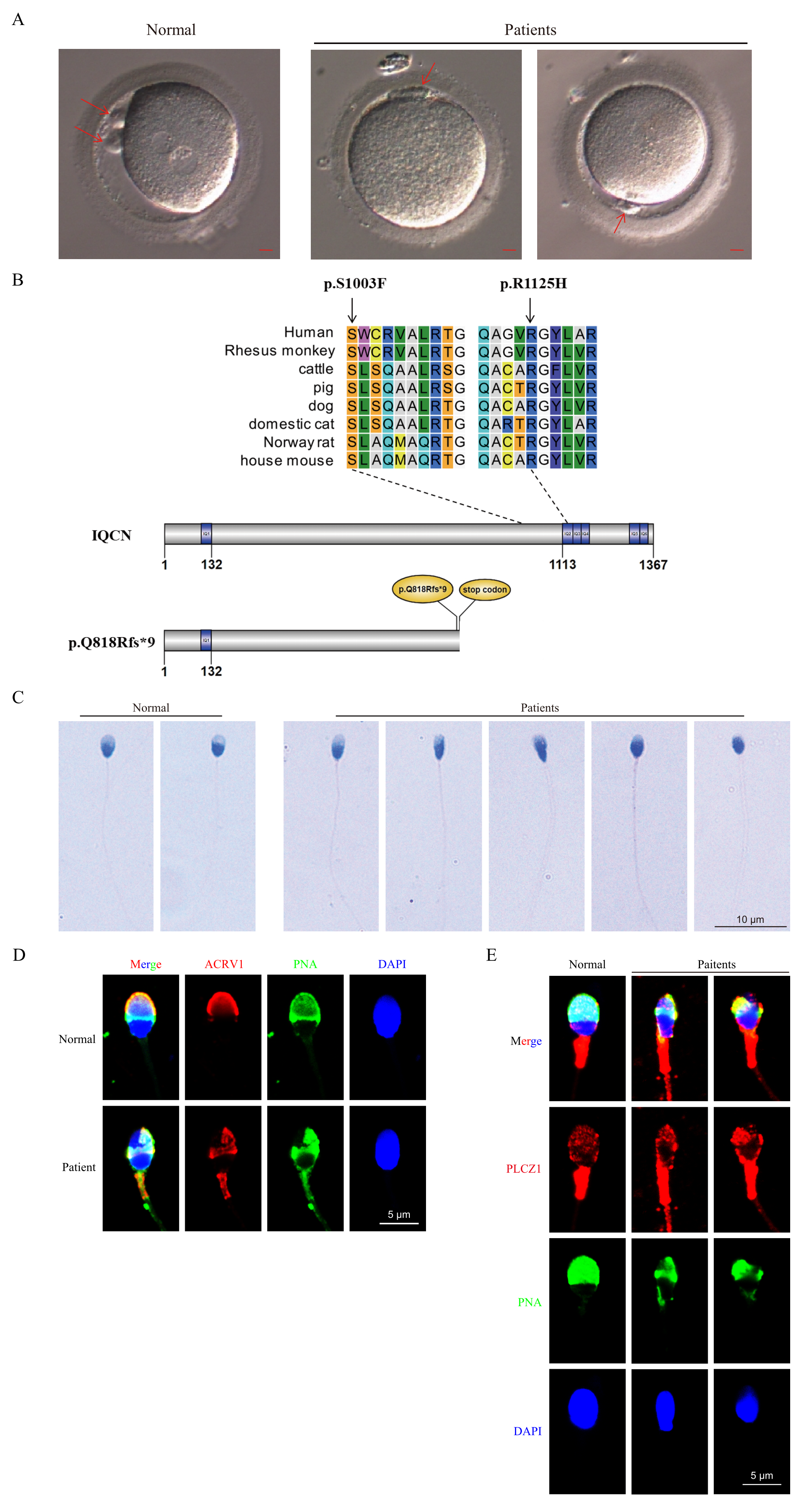


**Figure S1.** **Patients harboring *IQCN* variants exhibited fertilization failure and sperm acrosomal defects.**

(A) Normal oocytes could not be fertilized by spermatozoa from patients by In vitro fertilization (IVF) and Rescue-Intracytoplasmic Sperm Injection (R-ICSI). Two pronuclei and two polar bodies (red arrows) are found in normal fertilized oocytes, whereas only one polar body (red arrow) is observed in the unfertilized oocytes. Scale bars: 10 μm.

(B) Locations and conservation analysis of the detected variants in *IQCN.* Conservation of the missense sequence variants is indicated by the alignment of eight species. Both amino acid sites p.S1003 and p.R1125 are highly conserved according to the sequence alignment. The positions of the two missense variants are indicated in the protein structure of IQCN. Blue boxes represent the IQ domains. There are six IQ domains in the IQCN wild-type protein. The newly formed IQCN truncated protein p.Q818Rfs*9, which lost five IQ domains in the C-terminal of IQCN protein, is shown. All domains and regions are described by the Uniprot server.

(C) Morphology of spermatozoa from a fertile normal control and patients with biallelic *IQCN* variants. Spermatozoa from the fertile control show normal head shape, whereas spermatozoa from men with biallelic *IQCN* variants display tapered sperm head. Scale bars: 10 μm.

(D) Immunofluorescence staining of ACRV1 in ejaculated sperm from a fertile normal control and patient with biallelic *IQCN* variants. Anti-ACRV1 (red) antibody and PNA (green) were used. The nuclei of sperm are DAPI labeled (blue). Scale bars: 5 μm.

(E) Immunofluorescence staining of PLCZ1 in ejaculated sperm from a fertile normal control and patient with biallelic *IQCN* variants. Anti-PLCZ1 (red) antibody and PNA (green) were used. The nuclei of sperm are DAPI labeled (blue). The staining of PLCZ1 in the sperm flagella is non-specific. Scale bars: 5 μm.


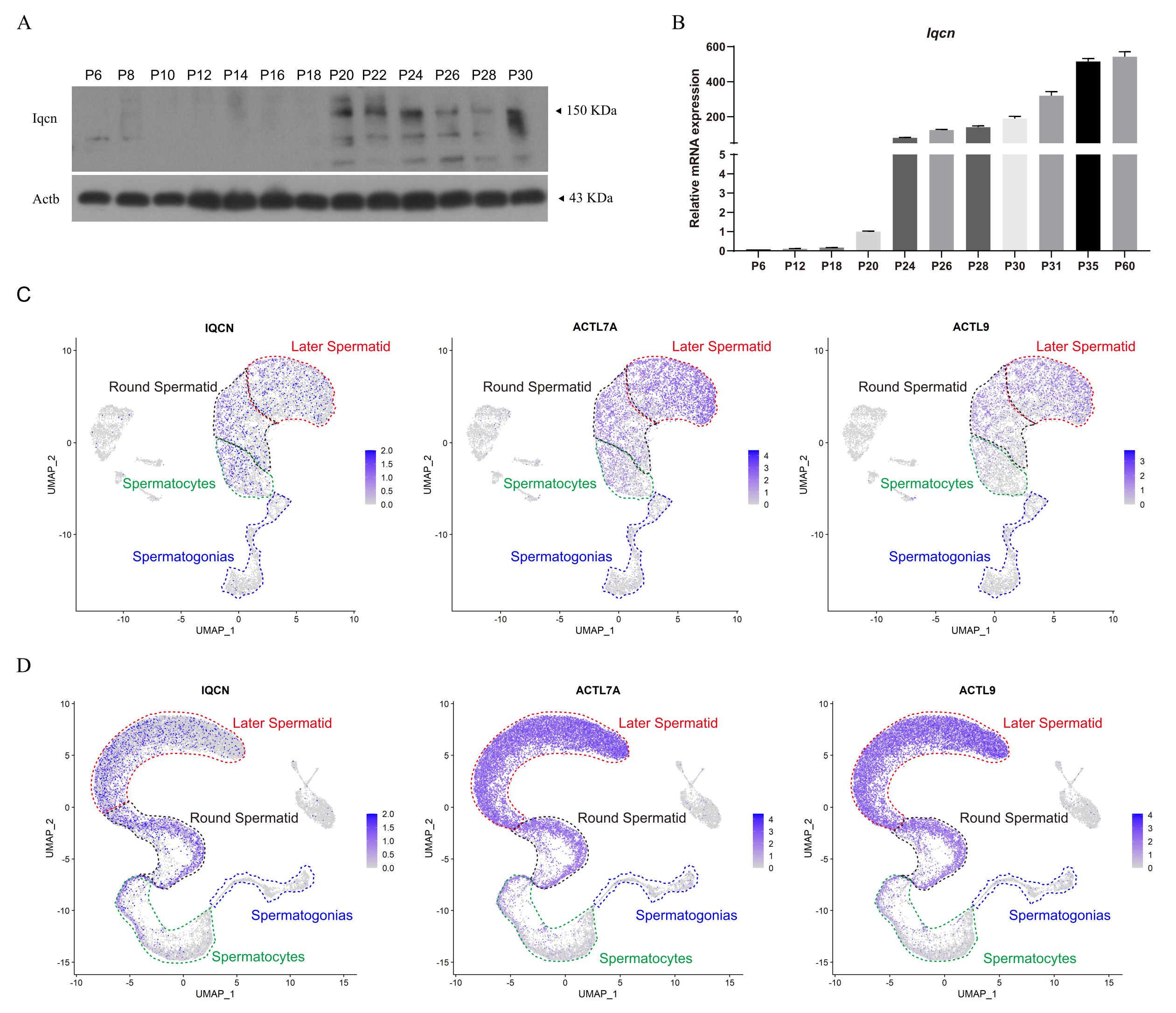


**Figure S2.** **Mouse Iqcn, human and monkey IQCN were expressed during spermiogenesis.**

(A) Western blot analysis shows mouse Iqcn expressed during the spermiogenesis stage after postnatal day 20 (P20). The 150 kDa band indicated the Iqcn protein. Actb was used as the reference protein.

(B) Mouse *Iqcn* is increasingly expressed after P20 by analyzing the mRNA level.

(C) UMAP plot of single cell RNA sequence (Data was obtained from GSE142585) analysis revealed that *IQCN* transcripts are mainly expressed in round and later stage spermatids in human, which is consistent with the expression of *Actl7a* and *Actl9* in humans. The color key from white to blue indicates low-to-high expression level.

(D) UMAP plot of single cell RNA sequence (Data was obtained from GSE142585) analysis reveals that *IQCN* transcripts is mainly expressed in round and later stage spermatids in monkey, which are consistent with the expression patterns of *Actl7a* and *Actl9* in monkeys. The color key from white to blue indicates low-to-high expression level.

UMAP, Uniform Manifold Approximation and Projection


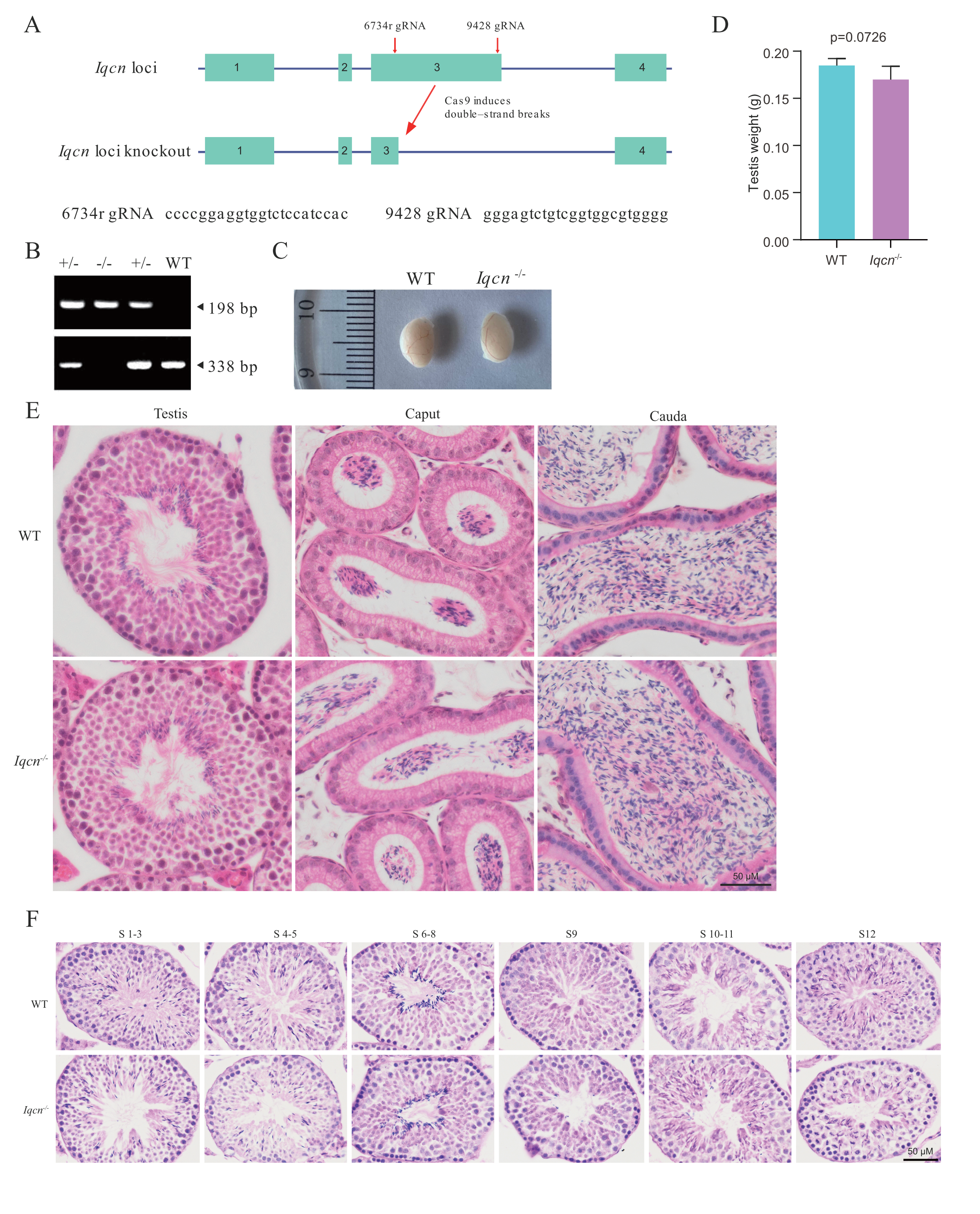


**Figure S3. Homozygous *Iqcn*^‒/‒^ mice were generated.**

(A) Generation of *Iqcn*^‒/‒^ mice. sgRNA design for targeting the *Iqcn* locus by CRISPR-Cas9.

(B) Genotyping to identify *Iqcn*^‒/‒^ mice. The 198 bp PCR product indicates the *Iqcn* mutant allele and the 338 bp PCR product indicates the *Iqcn* WT allele.

(C) Testis sizes are comparable between WT and *Iqcn*^‒/‒^ male mice at two-months-old.

(D) Testis weights are comparable between WT and *Iqcn*^‒/‒^ male mice at two-months-old.

(E) HE staining of testis, caput, and cauda from WT and *Iqcn*^‒/‒^ male mice. There is no significant pathological difference between WT and *Iqcn*^‒/‒^ male mice. Scale bars: 50 μm.

(F) PAS staining of step 1-12 spermatids from WT and *Iqcn*^‒/‒^ male mice testes. Based on visual observations, there is no marked pathological difference between WT and *Iqcn*^‒/‒^ male mice. Scale bars: 50 μm.

PCR, polymerase chain reaction; sgRNA, single guide RNA; WT, wild type; PAS, periodic acid–Schiff.


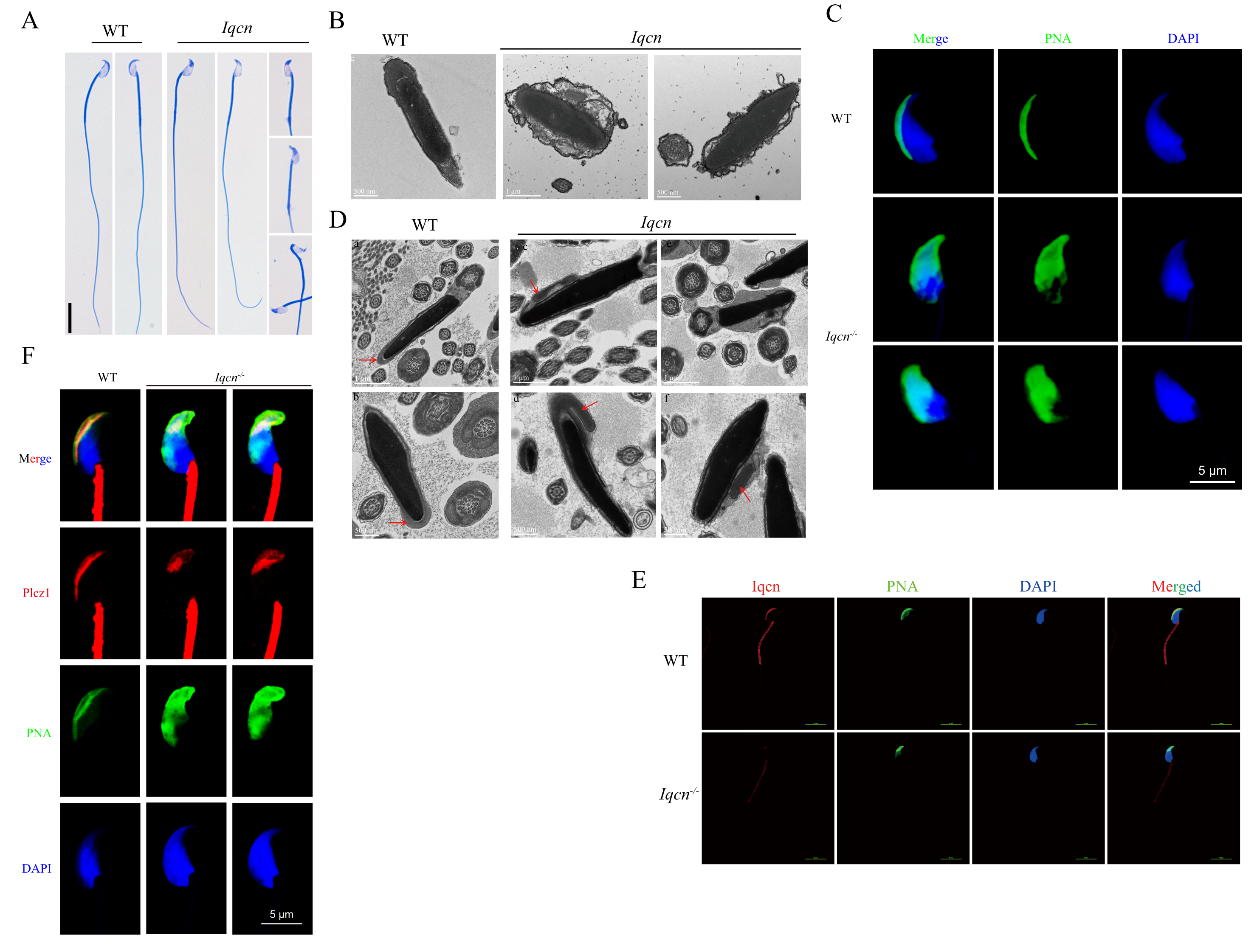


**Figure S4. Characterization of *Iqcn*^‒/‒^ male mice.**

(A) Morphology of mice spermatozoa from WT and *Iqcn*^‒/‒^ male mice. Spermatozoa from WT mice show normal head shape, whereas spermatozoa from *Iqcn*^‒/‒^ male mice display sperm head defects. Scale bars: 10 μm.

(B) Ultrastructure of ejaculated spermatozoa determined by TEM from WT and *Iqcn*^‒/‒^ male mice. No acrosomes are found in most ejaculated spermatozoa from *Iqcn*^‒/‒^ male mice, or only a small amount of acrosome detached from the nuclear envelope. Ac, acrosome. Scale bars: 500 nm and 1 μm.

(C) PNA staining of sperm acrosomes from WT and *Iqcn*^‒/‒^ male mice. Scale bars: 5 μm.

(D) Ultrastructure of sperm in the epididymis determined by TEM from WT and *Iqcn*^‒/‒^ male mice. The acrosome is found in sperm in the epididymis from *Iqcn*^‒/‒^ male mice, however, it is detached from the nuclear envelope. Red arrows indicate acrosomes. Scale bars: 500 nm and 1 μm.

(E) Immunofluorescence staining of Iqcn in sperm from WT and *Iqcn*^‒/‒^ male mice. Anti-Iqcn (red) antibody and PNA (green) were used. The nuclei of sperm are DAPI labeled (blue). Scale bars: 10 μm.

(F) Immunofluorescence staining of Plcz1 in sperm from WT and *Iqcn*^‒/‒^ male mice. Anti-Plcz1 (red) antibody and PNA (green) were used. The nuclei of sperm are DAPI labeled (blue). The staining of Plcz1 in the sperm flagella is non-specific. Scale bars: 5 μm.

DAPI, 4′,6-diamidino-2-phenylindole; PNA, peanut agglutinin; WT, wild type


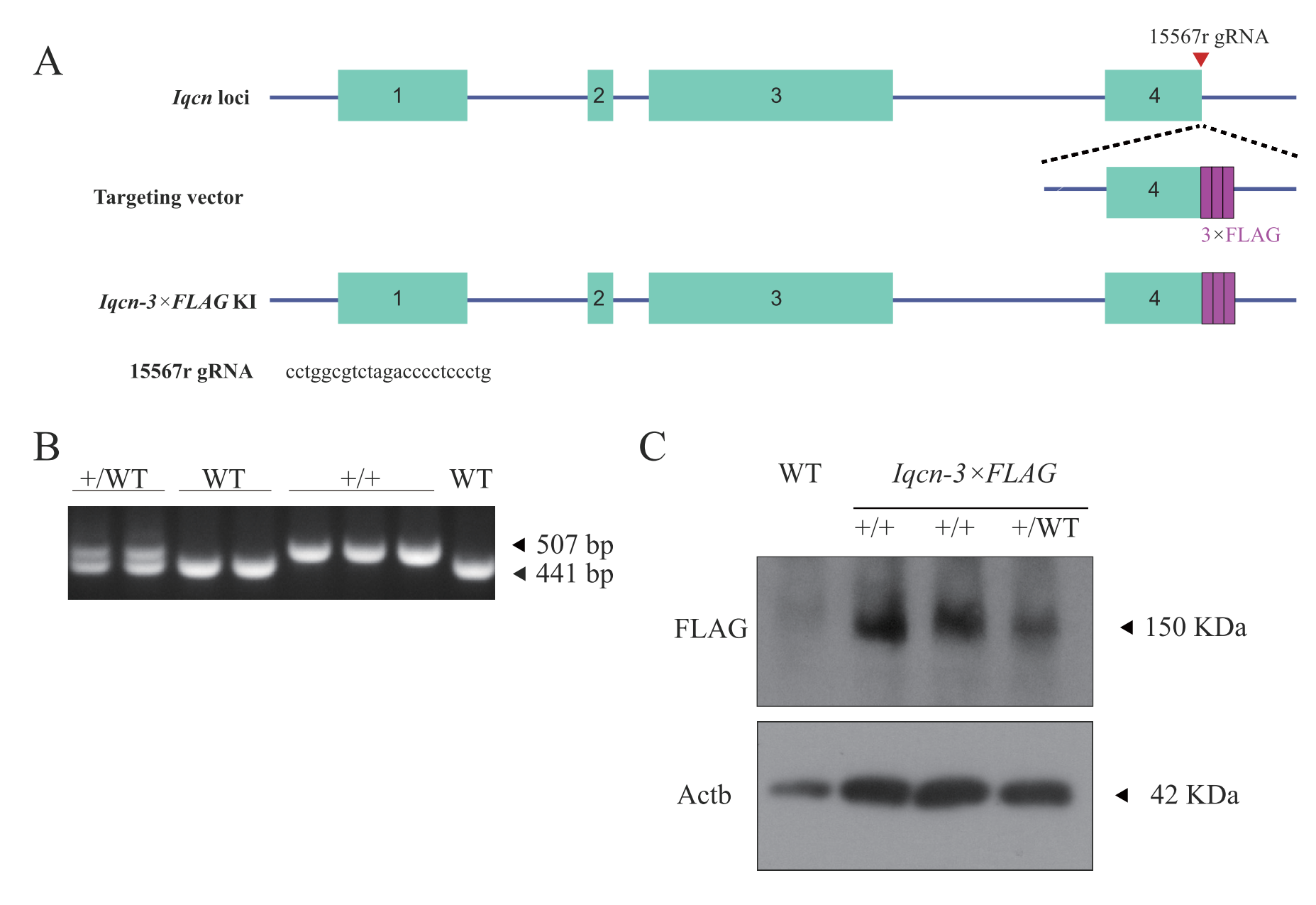


**Figure S5. Generation of *Iqcn-3×FLAG*^+/+^ mice.**

(A) Generation of *Iqcn-3×FLAG*^+/+^ mice. sgRNA design for targeting the *Iqcn* locus by CRISPR-Cas9.

(B) Genotyping to identify *Iqcn-3×FLAG*^+/+^ mice. The 507 bp PCR product indicates the *Iqcn-3×FLAG*^+/+^ allele and the 441 bp PCR product indicates the *Iqcn* WT allele.

(C) Western blot characterization of *Iqcn-3×FLAG*^+/+^ mice. Actb is the loading control.

PCR, polymerase chain reaction; sgRNA, single guide RNA


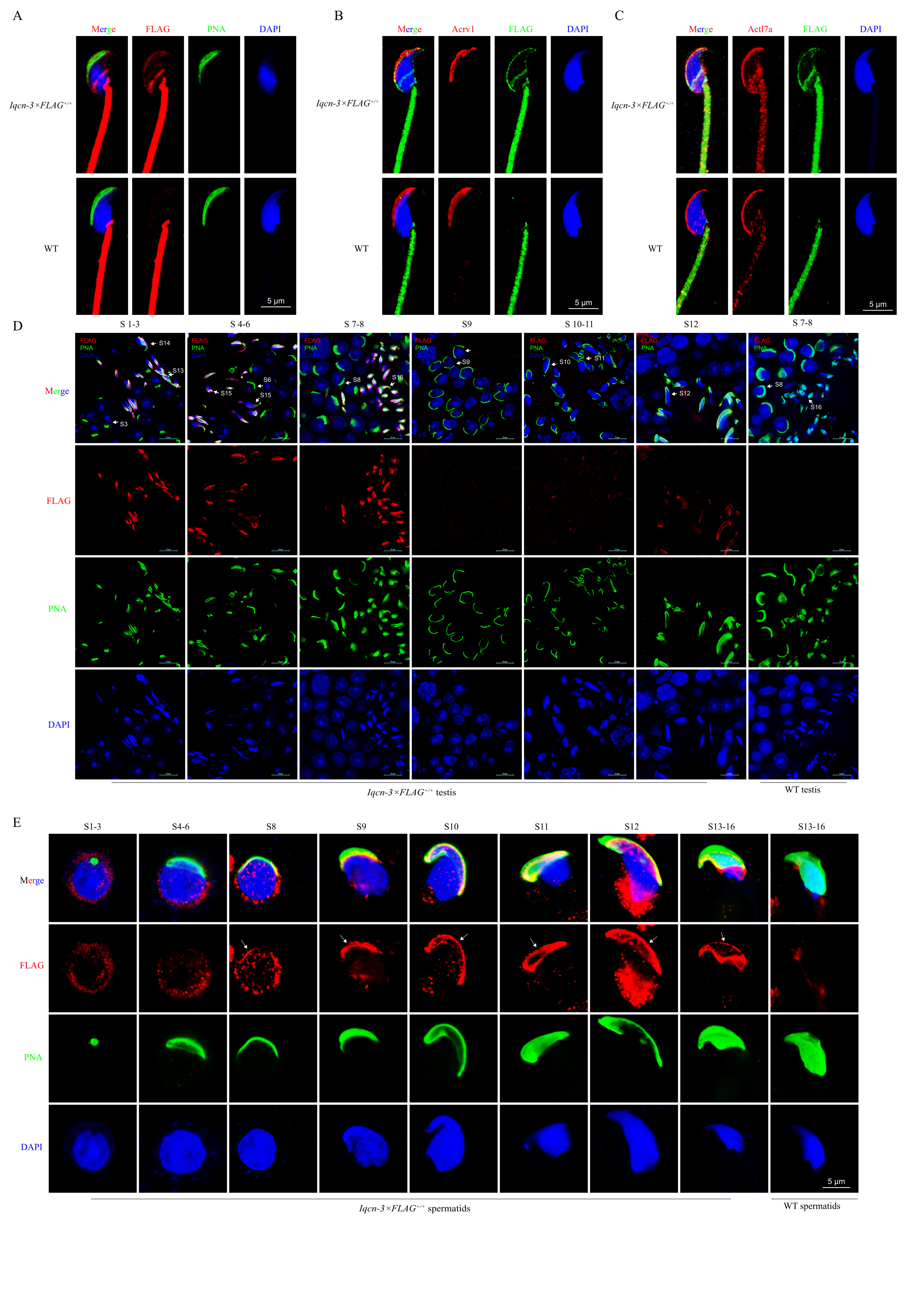


**Figure S6. Mouse Iqcn expression in the spermatozoa along the acroplaxome and predominantly in the marginal ring region.**

(A) Immunofluorescence staining of Iqcn in ejaculated sperm from *Iqcn-3×FLAG* homozygous knock-in mice. Anti-FLAG (red) antibody and PNA (green) were used. The nuclei of sperm are DAPI labeled (blue). The staining of FLAG-tag in the flagella is non-specific. *Iqcn-3×FLAG*^+/+^, *Iqcn-3×FLAG* homozygous knock-in mice; WT, wild-type mice. Scale bars: 5 μm.

(B) Co-immunofluorescence staining of Acrv1 and *Iqcn* in ejaculated sperm from *Iqcn-3×FLAG*^+/+^ mice. Anti-Acrv1 (red) antibody and anti-FLAG (green) were used. The nuclei of sperm are DAPI labeled (blue). The staining of FLAG-tag in the flagella is non-specific. Scale bars: 5 μm.

(C) Co-immunofluorescence staining of Actl7a and Iqcn in ejaculated sperm from *Iqcn-3×FLAG*^+/+^ mice. Anti-Actl7a (red) antibody and anti-FLAG (green) were used. The nuclei of sperm are DAPI labeled (blue). The staining of FLAG-tag in the flagella is non-specific. Scale bars: 5 μm.

(D) Immunofluorescence staining of Iqcn in testes from *Iqcn-3×FLAG*^+/+^ mice. Step 1-12 spermatids in testes were analyzed. Anti-FLAG (red) antibody and PNA (green) were used. The nuclei of sperm are DAPI labeled (blue). WT, wild-type mice. Scale bars: 10 μm.

(E) Immunofluorescence staining of Iqcn in step 1-16 (S1-16) spermatids isolated from the testis of *Iqcn-3×FLAG*^+/+^ mice. Anti-FLAG (red) antibody and PNA (green) were used. The nuclei of sperm are DAPI labeled (blue). S13-16 spermatids from WT mice are also stained as a negative control for FLAG staining. Non-specific FLAG staining is observed. Scale bars: 5 μm.

DAPI, 4′,6-diamidino-2-phenylindole; *Iqcn-3×*FLAG^+/+^, *Iqcn-3×*FLAG homozygous knock-in mice; PNA, peanut agglutinin; S4-16, step 4-16; S8-16, step 8 to 16; S13-16, step 13-16; WT, wild type


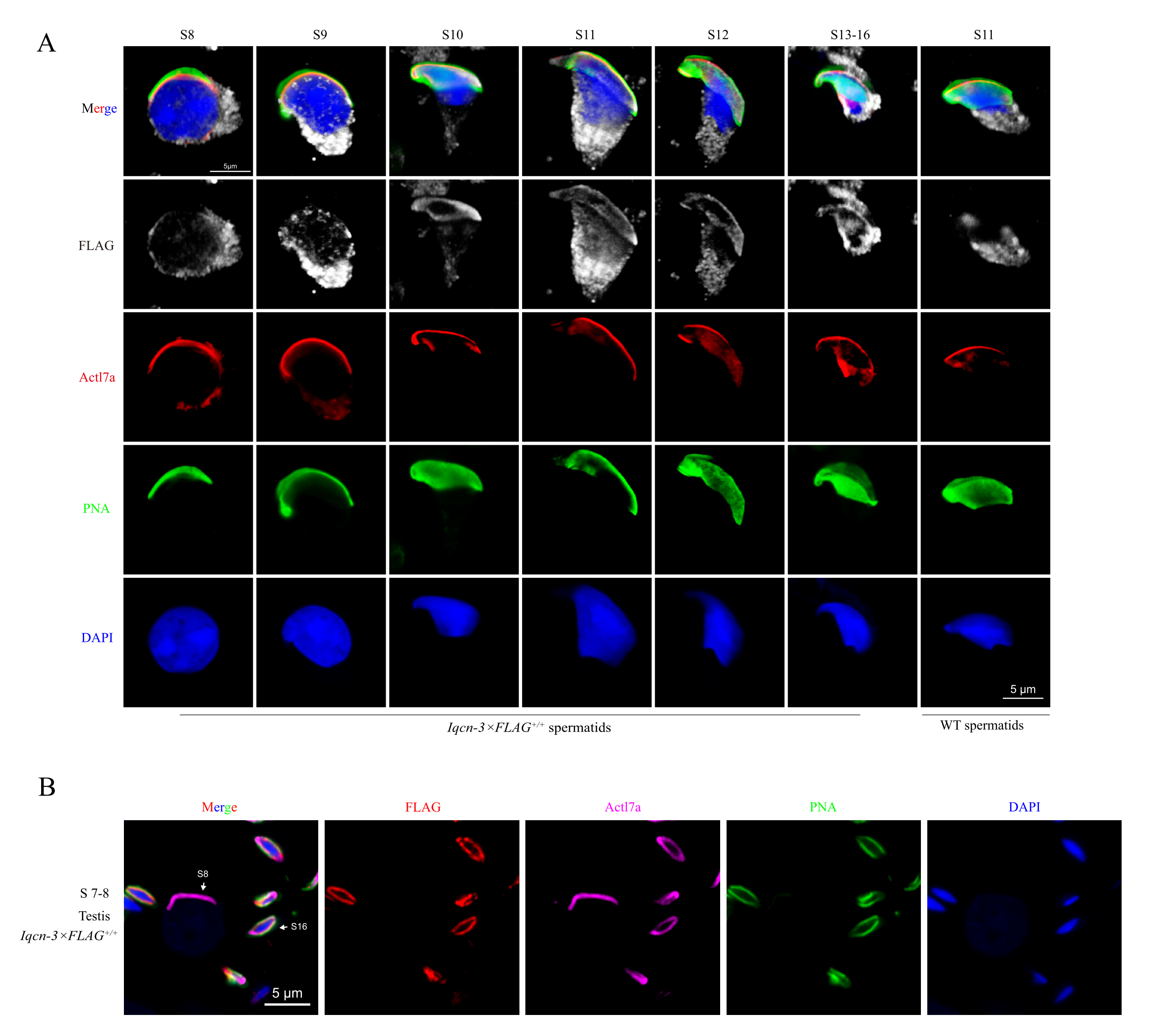


**Figure S7. Iqcn co-localized with Actl7a in the acroplaxome during mice spermiogenesis.**

(A) Co-immunofluorescence staining of Iqcn and Actl7a in S8-16 spermatids isolated from the testis of *Iqcn-3×FLAG*^+/+^ mice. Anti-FLAG (white) antibody, anti-Actl7a (red) antibody, and PNA (green) were used. The nuclei of sperm are DAPI labeled (blue). S11 spermatids from WT mice are also stained as a negative control for FLAG staining. Non-specific FLAG staining is observed. Scale bars: 5 μm.

(B) Co-immunofluorescence staining of Iqcn and Actl7a in spermatids from *Iqcn-3×FLAG*^+/+^ mice. Step 7-8 spermatids isolated from testes were analyzed. Anti-FLAG (red) antibody, anti-Actl7a (purple) antibody, and PNA (green) were used. The nuclei of sperm are DAPI labeled (blue). Scale bars: 5 μm.

DAPI, 4′,6-diamidino-2-phenylindole; PNA, peanut agglutinin; WT, wild type


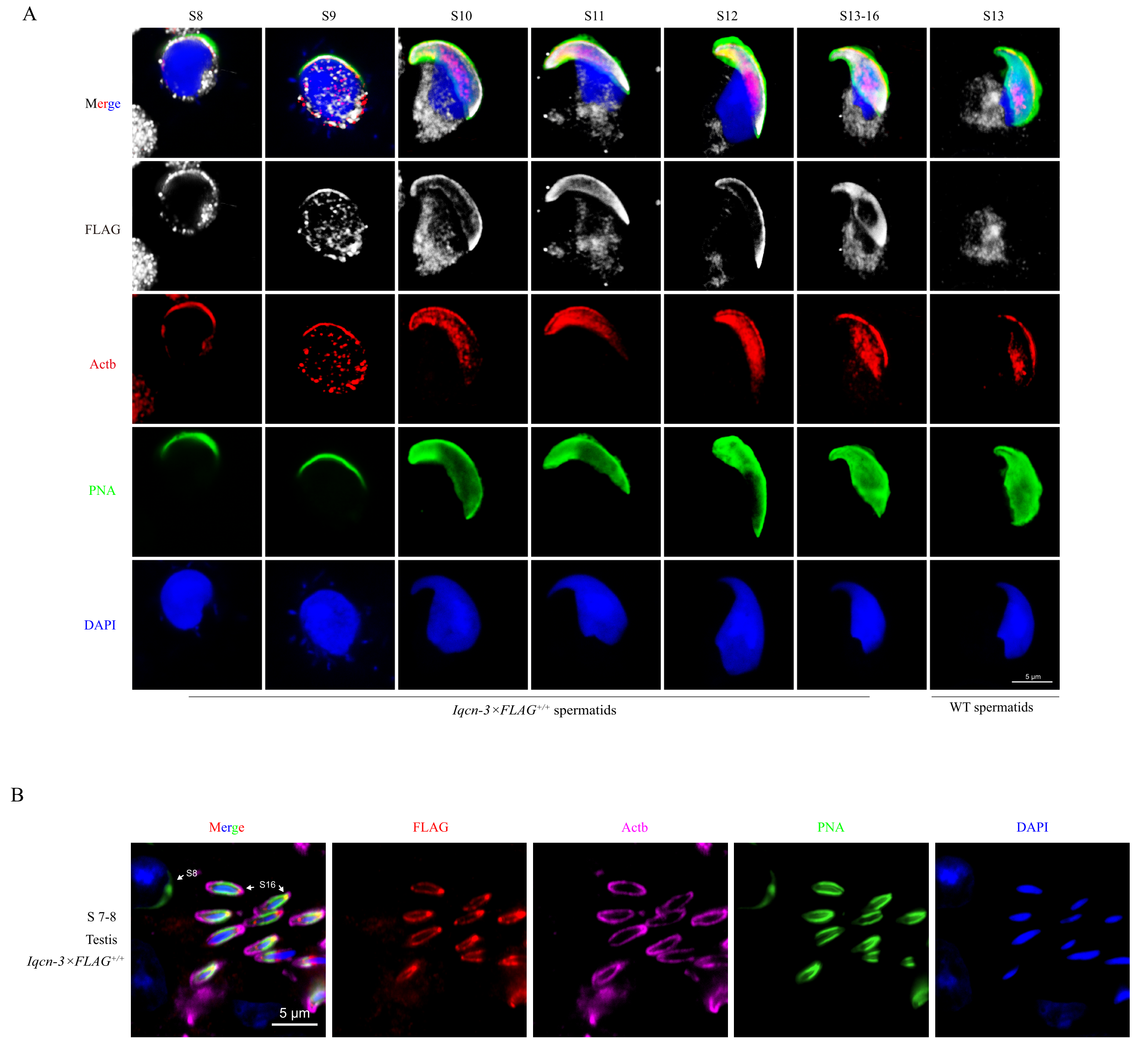


**Figure S8. Iqcn co-localized with Actb in the acroplaxome during mice spermiogenesis.**

(A) Co-immunofluorescence staining of Iqcn and Actb in Step 8-16 spermatids isolated from the testis of *Iqcn-3×FLAG*^+/+^ mice. Anti-FLAG (white) antibody, anti-Actb (red) antibody, and PNA (green) were used. The nuclei of sperm are DAPI labeled (blue). Step 13 spermatids from WT mice are also stained as a negative control for FLAG staining. Non-specific FLAG staining can be seen. Scale bars: 5 μm.

(B) Co-immunofluorescence staining of Iqcn and Actb in spermatids from *Iqcn-3×FLAG*^+/+^ mice. Step 7-8 spermatids isolated from testes were analyzed. Anti-FLAG (red) antibody, anti-Actb (purple) antibody, and PNA (green) were used. The nuclei of sperm are DAPI labeled (blue). Scale bars: 5 μm.

DAPI, 4′,6-diamidino-2-phenylindole; PNA, peanut agglutinin; WT, wild type


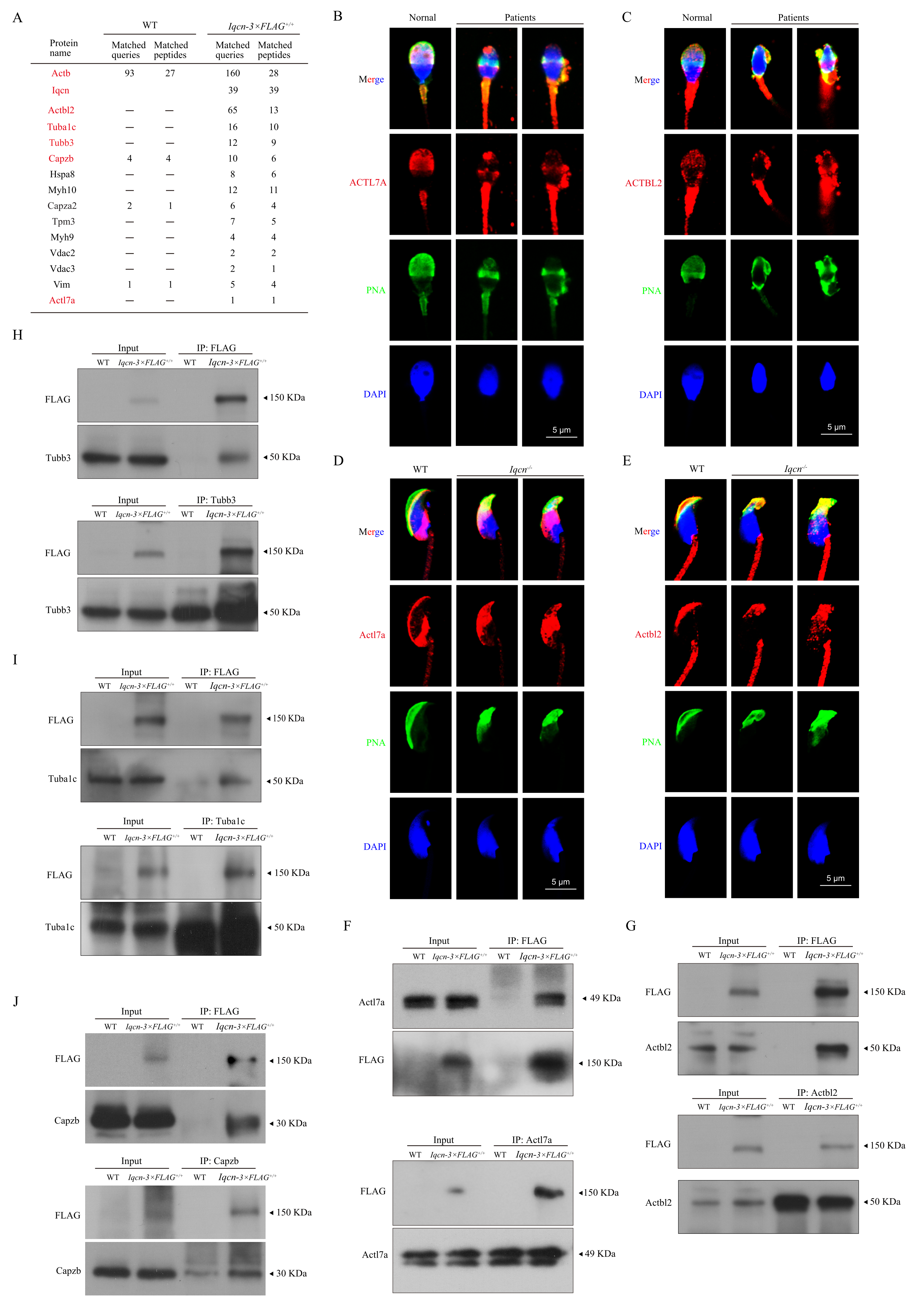


**Figure S9. Iqcn maintains acroplaxome integrity by interacting with many Actin-associated proteins including Actl7a and Actbl2.**

(A) Candidate proteins that interact with Iqcn. Mass spectrometry is used to identify the interaction proteins with Iqcn.

(B) Immunofluorescence staining of ACTL7A in spermatozoa from a fertile normal control and patients with biallelic *IQCN* variants. Anti-ACTL7A (red) antibody and PNA (green) were used. The nuclei of sperm are DAPI labeled (blue). Scale bars: 5 μm.

(C) Immunofluorescence staining of ACTBL2 in spermatozoa from a fertile normal control and patients with biallelic *IQCN* variants. Anti-ACTBL2 (red) antibody and PNA (green) were used. The nuclei of sperm are DAPI labeled (blue). Scale bars: 5 μm.

(D) Immunofluorescence staining of Actl7a in spermatozoa from WT and *Iqcn*^‒/‒^ male mice. Anti-Actl7a (red) antibody and PNA (green) were used. The nuclei of sperm are DAPI labeled (blue). Scale bars: 5 μm.

(E) Immunofluorescence staining of Actbl2 in spermatozoa from WT and *Iqcn*^‒/‒^ male mice. Anti-Actbl2 (red) antibody and PNA (green) were used. The nuclei of sperm are DAPI labeled (blue). Scale bars: 5 μm.

(F) Interaction between Iqcn and Actl7a following co-IP in sperm from *Iqcn-3×FLAG*^+/+^ mice. FLAG-tagged Iqcn interacts with Actl7a.

(G) Interaction between Iqcn and Actbl2 following co-IP in sperm from *Iqcn-3×FLAG*^+/+^ mice. FLAG-tagged Iqcn interacts with Actbl2.

(H) Interaction between Iqcn and Tubb3 following co-IP in sperm from *Iqcn-3×FLAG*^+/+^ mice. FLAG-tagged Iqcn interacts with Tubb3.

(I) Interaction between Iqcn and Tuba1c following co-IP in sperm from Iqcn-*3×FLAG^+/+^* mice. FLAG-tagged Iqcn interacts with Tuba1c.

(J) Interaction between Iqcn and Capzb following co-IP in sperm from Iqcn-*3×FLAG^+/+^* mice. FLAG-tagged Iqcn interacts with Capzb.

co-IP, co-immunoprecipitation; DAPI, 4′,6-diamidino-2-phenylindole; PNA, peanut agglutinin; WT, wild type


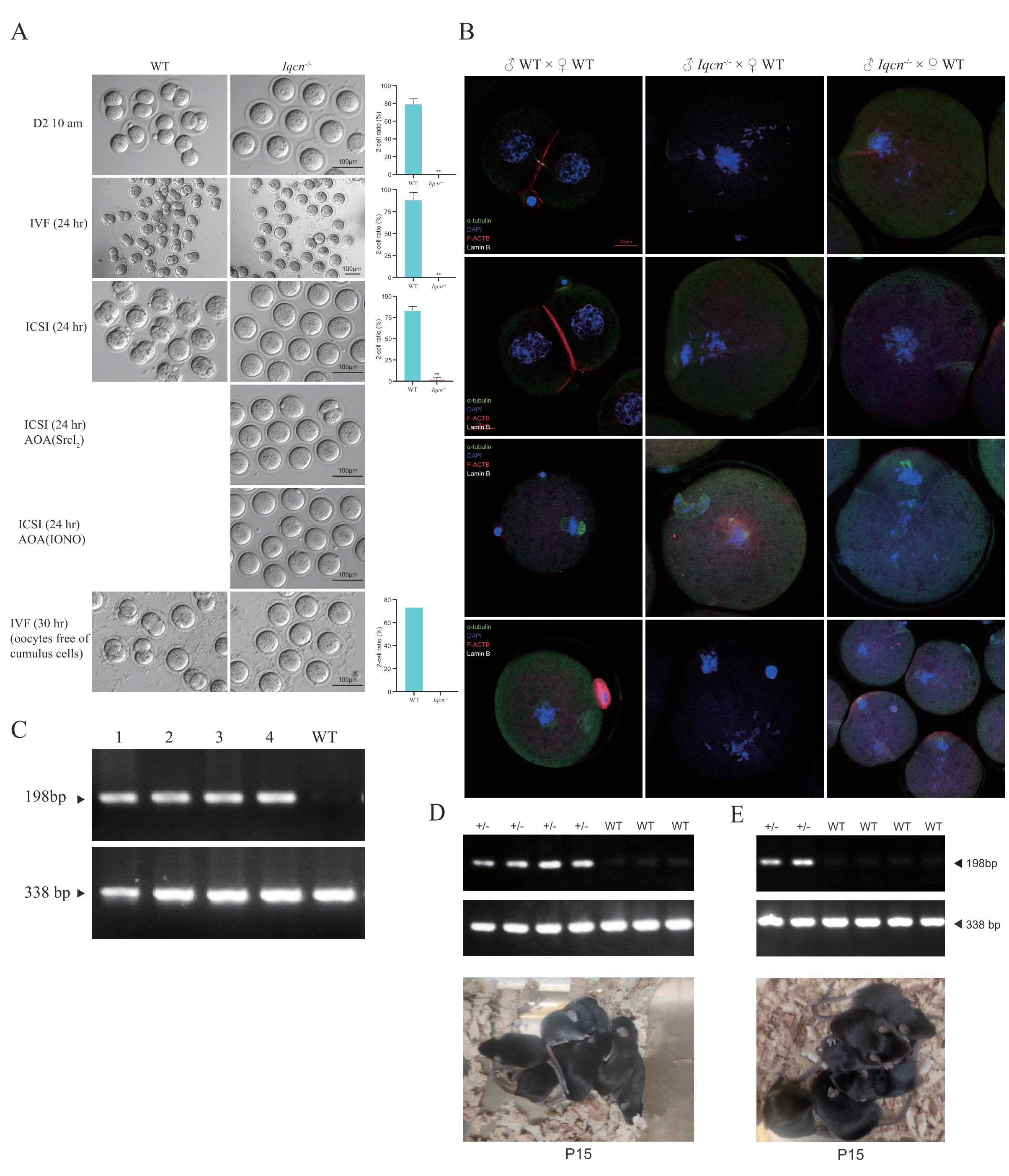


**Figure S10. Sperm from *Iqcn* knock-out mice failed to fertilize normal oocytes via IVF or ICSI, however, it fertilized the oocytes free of zona pellucida.**

(A) IVF, ICSI, and ICSI+AOA using SrCl_2_ or calcium ionophore ionomycin (IONO) failed to enable the sperm from *Iqcn*^‒/‒^ male mice to fertilize the normal oocytes. The third lane indicates the proportion of two-cell embryos using sperm from WT or *Iqcn*^‒/‒^ male mice with different fertilization methods. Almost no two-cell embryos developed using the sperm from *Iqcn*^‒/‒^ male mice. Bars indicate means ± SEM. ***p* <0.01. In the D2 experiment, 45 and 42 oocytes were used in WT and *Iqcn*^‒/‒^ group, respectively. In the IVF experiment, 80 and 85 oocytes were used in WT and *Iqcn*^‒/‒^ group, respectively. In the ICSI experiment, 48 and 50 oocytes were used in WT and *Iqcn*^‒/‒^ group, respectively. In the IVF (oocytes free of cumulus cells) experiment, 37 and 45 oocytes were used in WT and *Iqcn*^‒/‒^ group, respectively.

(B) ICSI was performed using sperm from WT and *Iqcn*^‒/‒^ male mice. Two pronuclei were well formed after ICSI treatment using WT sperm, whereas male pronuclei were poorly formed after ICSI treatment using sperm from *Iqcn*^‒/‒^ male mice. Anti-α-tubulin (green) antibody, anti-F-Actb (red) antibody, and anti-Lamin B (white) were used. The nuclei of sperm are DAPI labeled (blue). Scale bars: 20 μm.

(C) Genotyping to identify the offspring of *Iqcn*^‒/‒^ male mice born after fertilization of oocytes free of zona pellucida. The 198 bp PCR product indicates the *Iqcn* mutant allele and the 338 bp PCR product indicates the *Iqcn* WT allele.

(D) Genotyping to identify the offspring of *Iqcn*^‒/‒^ male mice born after fertilization of oocytes free of zona pellucida. The first experiment shows that four pups can normally develop to postnatal day 15 (P15).

(E) The second experiment shows that two pups can normally develop to P15. The 198 bp PCR product indicates the *Iqcn* mutant allele and the 338 bp PCR product indicates the *Iqcn* WT allele.

AOA, artificial oocyte activation; ICSI, Intracytoplasmic Sperm Injection; IONO, calcium ionophore ionomycin; IVF, in vitro fertilization; DAPI, 4′,6-diamidino-2-phenylindole; PCR, polymerase chain reaction**;** SEM, standard error of the mean; WT, wild type


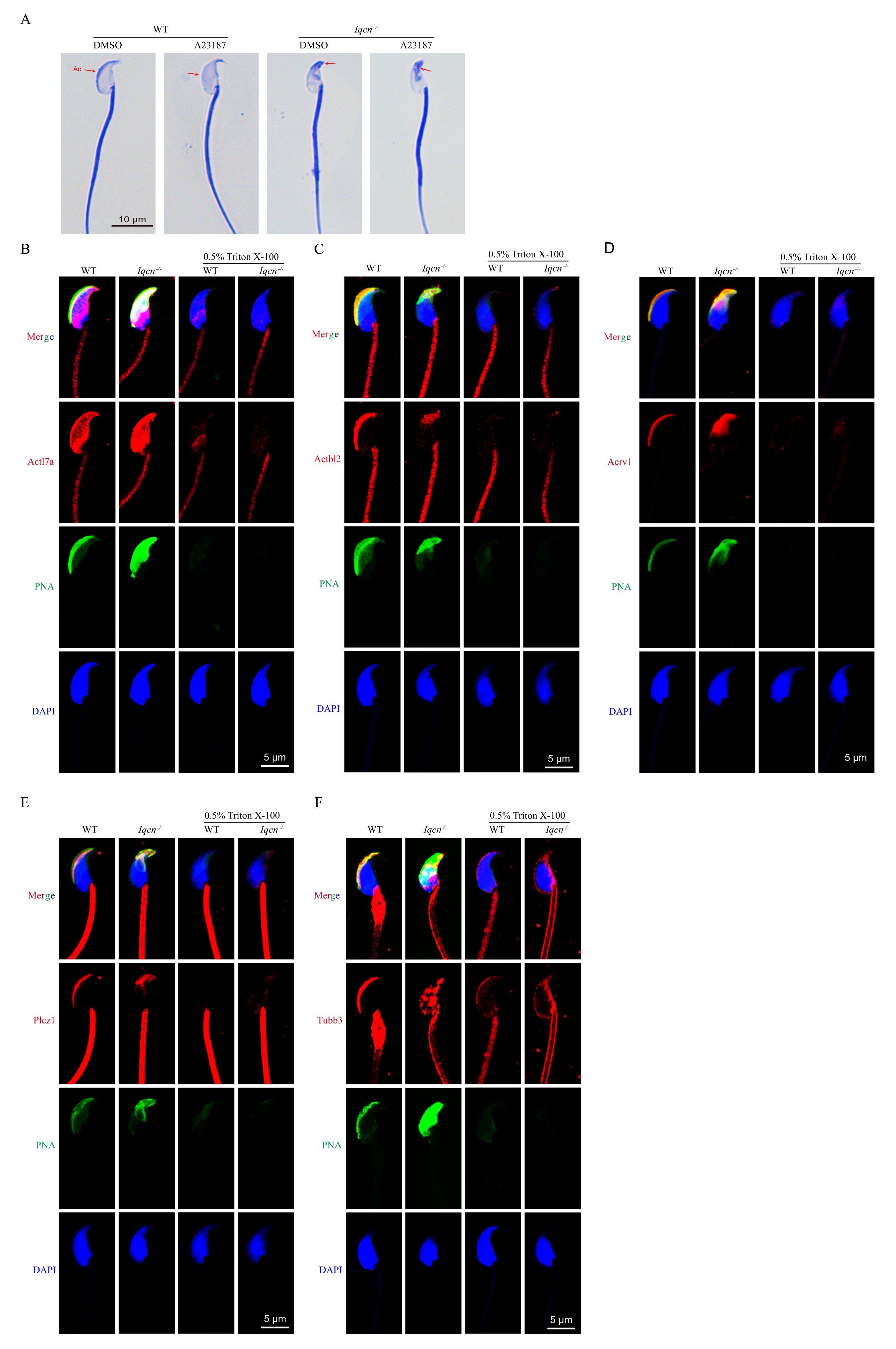


**Figure S11.** **The defective acrosome caused by *Iqcn* deficiency is the barrier to the success of ICSI treatment.**

(A) Coomassie blue staining shows changes in acrosomes after sperm capacitation by A23187 treatment in spermatozoa from WT. However, in spermatozoa from *Iqcn*^‒/‒^ male mice, the acrosome reaction is not induced by A23187 treatment. The red arrows point to the acrosome region. Scale bars: 10 μm.

(B-F) Immunofluorescence staining of Actl7a (B), Actbl2 (C), Acrv1 (D), Plcz1 (E), and Tubb3 (F) in spermatozoa from WT and *Iqcn*^‒/‒^ male mice after 0.5% Triton X-100 treatment. Anti-Actl7a, Actbl2, Acrv1, Plcz1, Tubb3 antibody (red) and PNA (green) were used, respectively. The nuclei of sperm are DAPI labeled (blue). Scale bars: 5 μm.

DAPI, 4′,6-diamidino-2-phenylindole; ICSI, Intracytoplasmic Sperm Injection; WT, wild type


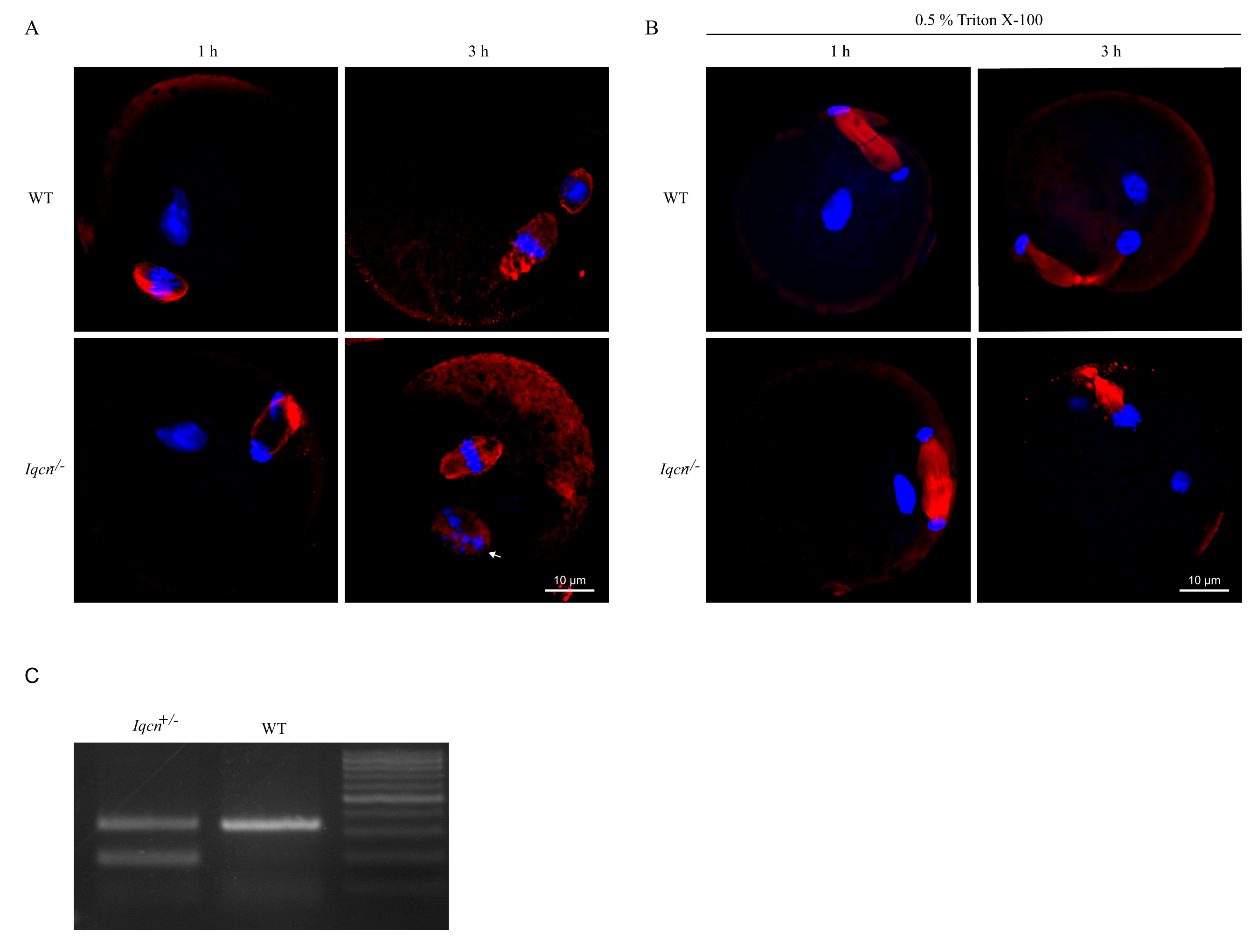


**Figure S12. Normal oocytes could be fertilized** **via ICSI followed by AOA treatment using** ***Iqcn*^‒/‒^ sperm without acrosome.**

(A) *Iqcn*^‒/‒^ male pronuclei do not form after ICSI treatment. Anti-α-tubulin antibody (red) was used. The nuclei of female and male are DAPI labeled (blue). The white arrow indicates the poorly formed pronuclei. Scale bars: 10 μm.

(B) *Iqcn*^‒/‒^ male pronuclei could form after ICSI followed by AOA treatment of the *Iqcn*^‒/‒^ sperm without acrosome. Anti-α-tubulin antibody (red) was used. The nuclei of female and male are DAPI labeled (blue). Sperm from both WT and *Iqcn*^‒/‒^ male mice were treated with 0.5% Triton X-100 to remove the acrosome. Scale bars: 10 μm.

(C) Genotyping to identify one pup of *Iqcn*^‒/‒^ sperm without acrosome after fertilization of oocytes.

AOA, artificial oocyte activation; DAPI, 4′,6-diamidino-2-phenylindole; ICSI, Intracytoplasmic Sperm Injection; WT, wild type
